# Gaining Mechanistic Insight Into Coproporphyrin I as Endogenous Biomarker for OATP1B‐Mediated Drug–Drug Interactions Using Population Pharmacokinetic Modeling and Simulation

**DOI:** 10.1002/cpt.983

**Published:** 2018-01-17

**Authors:** Shelby Barnett, Kayode Ogungbenro, Karelle Ménochet, Hong Shen, Yurong Lai, W. Griffith Humphreys, Aleksandra Galetin

**Affiliations:** ^1^ Centre for Applied Pharmacokinetic Research University of Manchester UK; ^2^ Investigative ADME, UCB Slough UK; ^3^ Pharmaceutical Candidate Optimization, Bristol‐Myers Squibb Princeton New Jersey USA; ^4^ Present address: Current affiliation for Yurong Lai: Gilead Science Foster City California

## Abstract

This study evaluated coproporphyrin I (CPI) as a selective endogenous biomarker of OATP1B‐mediated drug–drug interactions (DDIs) relative to clinical probe rosuvastatin using nonlinear mixed‐effect modeling. Plasma and urine CPI data in the presence/absence of rifampicin were modeled to describe CPI synthesis, elimination clearances, and obtain rifampicin *in vivo* OATP Ki. The biomarker showed stable interoccasion baseline concentrations and low interindividual variability (<25%) in subjects with wildtype *SLCO1B1*. Biliary excretion was the dominant CPI elimination route (maximal >85%). Estimated rifampicin *in vivo* unbound OATP Ki (0.13 μM) using CPI data was 2‐fold lower relative to rosuvastatin. Model‐based simulations and power calculations confirmed sensitivity of CPI to identify moderate and weak OATP1B inhibitors in an adequately powered clinical study. Current analysis provides the most detailed evaluation of CPI as an endogenous OATP1B biomarker to support optimal DDI study design; further pharmacogenomic and DDI data with a panel of inhibitors are required.

**Study Highlights**

**☑ WHAT IS THE CURRENT KNOWLEDGE ON THE TOPIC?**

CPI is proposed as a promising endogenous biomarker for assessing OATP1B‐mediated DDIs, based on increased plasma exposure following administration of a strong OATP inhibitor, rifampicin.

**☑ WHAT QUESTION DID THIS STUDY ADDRESS?**

A semimechanistic model was developed to evaluate CPI as an endogenous OATP1B biomarker and its synthesis, elimination routes, and selectivity. Comparison of CPI and rosuvastatin as probes was also conducted through estimation of rifampicin *in vivo* OATP Ki.

**☑ WHAT THIS STUDY ADDS TO OUR KNOWLEDGE**

This is the first study to estimate the synthesis and elimination of an endogenous OATP1B biomarker CPI using a modeling approach. The model developed was applied to assess sensitivity of CPI to identify moderate and weak OATP1B inhibitors and perform power calculations to guide optimal clinical DDI study design.

**☑ HOW THIS MIGHT CHANGE CLINICAL PHARMACOLOGY OR TRANSLATIONAL SCIENCE**

Modeling and simulation presented the utility of CPI as a selective endogenous biomarker for investigating weak to potent OATP1B‐mediated DDIs in adequately powered clinical DDI study.

Organic anion transporting polypeptides (OATP) 1B1 and 1B3 play a crucial role in the hepatic uptake of a variety of drugs and are associated with numerous drug–drug interactions (DDIs).^1^, ^2^, ^3^, ^4^, ^5^ In recent years there is an increasing interest in identifying suitable endogenous biomarkers for investigation of transporter function and transporter‐mediated DDI risk in early drug development.^4^, ^6^, ^7^ Such biomarker data, in conjunction with modeling and simulation, would lead to improved prioritization and informed design of subsequent DDI studies with clinical probes and allow simultaneous investigation of multiple transporters. Although use of endogenous biomarkers has many potential advantages (e.g., assessment of complex DDIs, evaluation of the interaction risk in patient populations), this approach is associated with a number of challenges, as summarized recently.^4^, ^7^

Several endogenous biomarkers have been proposed for the assessment of OATP1B‐mediated DDIs, including bilirubin, coproporphyrins, bile acids, and their respective sulfate conjugates.^8^, ^9^, ^10^, ^11^ The majority of these studies have been conducted in preclinical species, in most cases in cynomolgus monkey, whereas a paucity of data has been reported in human.^6^, ^11^, ^12^ In addition, their utility for the prediction of OATP1B DDIs has not been thoroughly investigated.

A recent study by Lai *et al*.^6^ investigated the utility of heme synthesis byproducts coproporphyrin I (CPI) and coproporphyrin III (CPIII) for the evaluation of OATP1B‐mediated DDIs. The study reported comparable mean AUC ratios (AUCR) of CPI and clinical probe rosuvastatin (RSV) in the presence of a single dose of a reference OATP inhibitor, rifampicin (RIF).^6^ Despite these promising findings, understanding of CPI selectivity, sensitivity, and rates of its synthesis/elimination is crucial for its qualification as a biomarker.^4^, ^7^ Furthermore, the impact of factors such as optimal clinical study design and interindividual variability in baseline plasma concentrations of the endogenous biomarker need to be understood. Most of CPI is produced in erythrocytes in a multistep synthesis process; initial step catalyzed by ALA‐synthase has been indicated as a rate‐limiting step.^13^ Reported *in vitro* data in transfected cell lines suggest selectivity of CPI for OATP1B1/1B3 and that it is not a substrate of renal uptake transporters.^6^, ^11^, ^14^ In contrast, CPIII is also an OATP2B1 substrate and the involvement of renal uptake transporters has been suggested.^6^, ^14^

To verify the utility of CPI as an endogenous biomarker of OATP1B‐mediated DDIs, this study aimed to: 1) Characterize the synthesis and elimination of CPI in humans using population pharmacokinetic (PK) modeling of reported CPI plasma and urine data in the absence and presence of prototypical strong OATP inhibitor RIF; 2) Use clinical data to estimate *in vivo* OATP Ki values of RIF using CPI and the clinically relevant probe RSV; 3) Perform *in vitro* inhibition studies with RIF in human hepatocytes using CPI and RSV as OATP1B probes and compare those to *in vivo* estimates; 4) Perform simulations to assess sensitivity of CPI as an endogenous biomarker to identify DDI risk with moderate (2 < AUCR < 5) and weak (AUCR <2) OATP1B inhibitors; and 5) Perform power calculations to support optimal clinical DDI study design with CPI as an OATP1B DDI biomarker.

## RESULTS

### Analysis of individual clinical CPI data

**Figure 1:**
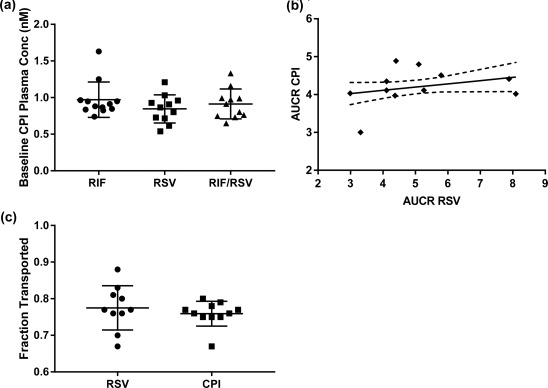
**(a)** Baseline plasma concentrations of coproporphyrin I taken prior to administration of rosuvastatin and/or rifampicin on three separate occasions (OCC1‐3). **(b)** Correlation between CPI and rosuvastatin AUCR in the presence of rifampicin. Data obtained in the same individuals.^6^ The solid line represents the line of linear regression and the dashed line represents the 95% confidence interval. **(c)** Individual calculated fT for rosuvastatin and CPI using rifampicin interaction data (Equation 1).

Analysis of baseline CPI plasma concentrations demonstrated low variability between subjects (<25% CV) and no significant differences between the three occasions (**Figure** 1 **a**). Comparison of the individual AUCR between CPI and RSV following RIF administration resulted in no significant correlation between probes (**Figure** 1 **b**), despite comparable estimated mean fraction eliminated via transporters (fT) (0.79 and 0.76 for RSV and CPI, respectively). Less pronounced between‐subject variability (13% CV) in DDI magnitude was evident for CPI in contrast to RSV (30% CV), reflected also in a wider range of estimated RSV fT (0.66–0.88, **Figure** 1 **c**).

### Structural PK models for CPI, RSV, and RIF

**Figure 2:**
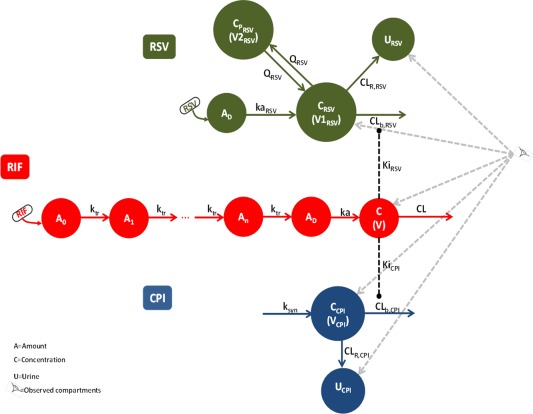
Schematic representation of the population PK models for rifampicin, coproporphyrin I (CPI), and rosuvastatin (RSV) and corresponding interactions of two probes with rifampicin (RIF).

**Figure 3:**
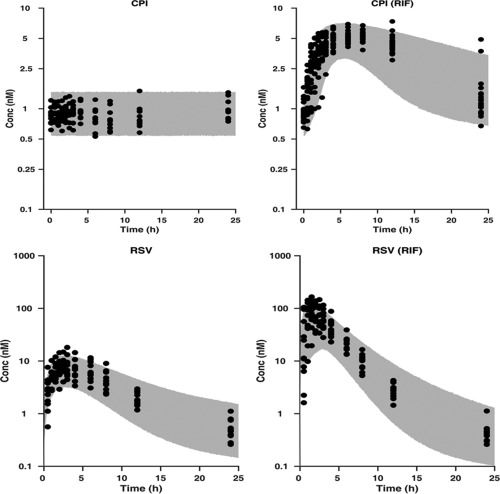
Upper panel: Visual predictive check of the developed population PK model for CPI plasma data, superimposed with the observed data. CPI and CPI (RIF) represent control and rifampicin phase of the study, respectively. Lower panel: Visual predictive check of the developed population PK model for RSV plasma data, superimposed with the observed data. RSV and RSV (RIF) represent control and rifampicin phase of the study, respectively. The gray area represents the 95% prediction of the simulated data and the dark circles are the observed data.

**Figure 4:**
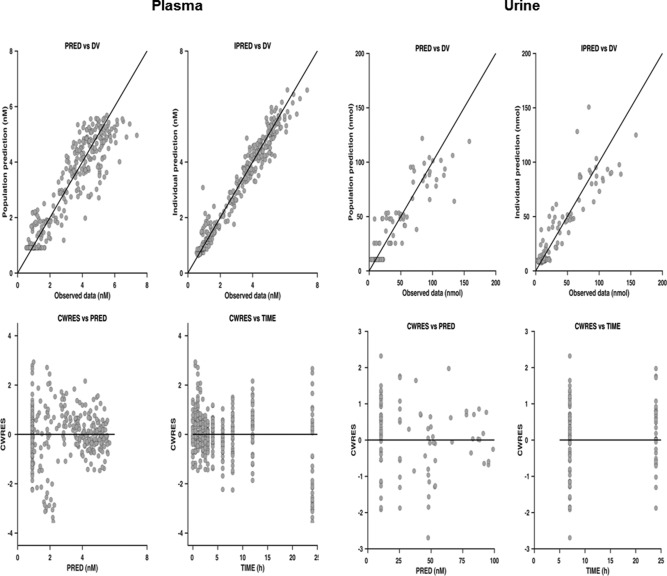
Goodness‐of‐fit plots for population PK model describing CPI plasma and urine data. DV, PRED, IPRED, and CWRES are the observed data, population and individual model prediction, and conditional weighted residuals, respectively.

The structural models used to describe plasma concentrations of perpetrator drug RIF and plasma and urine data for both OATP1B probes (CPI and RSV) are shown in **Figure** 2 **;** the corresponding parameter estimates obtained from sequential analysis are shown in **Table** 1 **.** The models that best described the plasma and urine data for RIF, CPI, and RSV were a one‐compartment first‐order elimination model with a transit absorption, a turnover model, and a two‐compartment first‐order absorption model, respectively. To assess the robustness of the model parameters and ability of the model to describe the observed data and variability adequately, standard visual predictive checks (VPC) and goodness of fit (GOF) plots were obtained for each of the individual models using respective plasma and urine data, as shown in **Figures** **3**, **4** (CPI) and in **Supplemental Figures S5, S6** (RIF and RSV).

**Table 1:** Parameter estimates of the population PK model for rifampicin, coproporphyrin I, and rosuvastatin

Drug	Parameter	Estimate (SE%)		
		Population	IIV	IOV
RIF^a^	ka (h^−1^)	2.09	46	49.2
	CL (L/h)	3.97	30.7	—
	V (L)	24.7	19.5	6.6
	MTT (h)	0.74	69.1	47.6
	*n*	8.63	24.3	—
	σ_prop_ (%)	31.3	—	—
	σ_add_ (μM)	0.01 (FIXED)	—	
CPI	k_syn_ (nM/h)	12.7 (6)	2.9 (318.7)	5.5 (45.6)
	CL_b,CPI_ (L/h)	12.3 (10)	14.3 (63.8)	18.3 (38)
	CL_R,CPI_ (L/h)	1.64 (6)	9.5 (31.9)	—
	V_CPI_ (L)	6.59 (12)	35.2 (24.7)	—
	V_CPI_ (L) (RIF)	3.4 (13)		—
	Ki_CPI_ (μM)^c^	1.15 (9)	18.8 (40.6)	—
	σ_prop_ (%) ‐ plasma	13.9 (10)	—	—
	σ_add_ (nM) ‐ plasma	0.001 FIXED	—	—
	σ_prop_ (%) ‐ urine	34.2 (6)	—	—
	σ_add_ (nM) ‐ urine	2.69 (28)	—	—
RSV	ka_RSV_ (h^−1^)	0.287 (9)	15.4 (57)	17.7 (42.7)
	CL_R,RSV_ (L/h)	8.48 (14)	37.9 (28.8)	—
	CL_b,RSV_ (L/h)	124 (11)	34.4 (22)	—
	V1_RSV_ (L)	430 (12)	21.8 (48.4)	—
	V1_RSV_ (L) (RIF)^b^	2.98 (50)		
	Q_RSV_ (L/h)	45.3 (19)	45.2 (34.4)	—
	Q_RSV_ (L/h) (RIF)^b^	5.03 (18)		
	V2_RSV_ (L)	865 (37)	—	—
	V2_RSV_ (L) (RIF)^b^	128 (42)		
	Ki_RSV_ (μM)^c^	2.23 (15)	33.4 (34.5)	—
	σ_prop_ (%) ‐ plasma	0.257 (6)	—	—
	σ_add_ (nM) ‐ plasma	0.05 FIXED	—	—
	σ_prop_ (%) ‐ urine	0.578 (16)	—	—
	σ_prop_ (nM) ‐ urine	0.1 FIXED	—	—

a NONMEM covariance step required for estimation of SE% failed.

b Parameter estimates for RSV when co‐administered with RIF.

c Value represents the total Ki estimated by the model. Correction for rifampicin unbound fraction in plasma of 0.11 reported in the clinical study (6), resulted in Ki values of 0.13 and 0.25 µM for CPI and RSV, respectively.

### Rifampicin population PK model

Parameter estimates of the population PK model for rifampicin are listed in **Table** 1 **.** The residual error was described by a combined proportional and additive residual error model. Interindividual variability (IIV) was estimated for all parameters; the estimates were less than 35% with the exception of ka and MTT (46% and 69%, respectively). Interoccasion variability (IOV) was estimated for ka, V, and MTT; the estimates were low for V (7%) and moderate for ka (49%) and MTT (48%). Despite substantial variability in the RIF data, especially at the absorption phase, the model adequately described the observed data and captured variability observed (**Figures S3, S7, S8**).

### CPI population PK model

The CPI structural model allowed estimation of biliary and renal components of its clearance. The analysis showed that biliary clearance is the predominant route of CPI elimination, with a maximal contribution of 88% to its total clearance (**Table** 1). The model implemented inhibition of *CL*_*b,CPI*_ by RIF in a competitive manner, with no effect of RIF on *CL*_*R,CPI*_ and CPI synthesis rate. The estimated unbound RIF Ki was 0.13 μM (based on measured RIF fraction unbound in plasma of 0.11^6^). The residual error for plasma and urine data were described by a combined proportional and additive residual error models with the additive component of plasma fixed. The population (fixed effects) parameter estimates of this model were well estimated (SE% less than 15%). IIV was estimated for all parameters (<35%) and the SE% were moderate (between 25 and 75%), with the exception of synthesis rate (*k*_*syn*_) (319%). IOV was estimated for *CL*_*b,CPI*_ (18%) and *k*_*syn*_ (6%). In the presence of RIF, estimated *V*_*CPI*_ was reduced by ∼50%. The CPI model adequately described the plasma and urine data and its variability under baseline and RIF condition (**Figures** 3 **,** 4 **, Figures S9–11**). Identifiability analysis showed that the structural model for CPI (**Figure** 2) was globally identifiable.

### Rosuvastatin population PK model

The structural model used to describe RSV plasma and urine data allowed estimation of clearance parameters by biliary (*CL*_*b,RSV*_) and renal (*CL*_*R,RSV*_) routes of elimination; biliary clearance was the predominant route (94%). Combined proportional and additive residual error models were used to describe the residual errors for both plasma and urine data. The additive components were fixed for plasma and urine data. Similar to CPI, plasma concentration and amount excreted in urine were increased when RSV was coadministered with RIF. Analogous to CPI, the model included RIF transporter inhibition constant (*Ki*_*RSV*_) for the biliary elimination of RSV to account for this; the unbound parameter value based on RSV data was 0.25 μM, comparable to RIF *in vivo* Ki estimated from pitavastatin and pravastatin clinical data (0.23 and 0.19 μM, respectively).^15^

RSV distribution parameters of the model (*V1*_*RSV*_, *V2*_*RSV*_, and *Q*_*RSV*_, estimated by inclusion of a binary covariate on these parameters) changed significantly (likelihood ratio test *p* < 10^−6^) in the presence of RIF (>90% reduction for *V1*_*RSV*_). This more pronounced effect on the distribution relative to CPI was also reflected in more evident change in RSV C_max_ (13.2‐fold increase), whereas changes in CPI AUC and C_max_ following RIF administration were comparable. IIV variability (≤45%) was estimated for all parameters except for *V2*_*RSV*_, with SE% <60%. IOV was estimated for *ka*_*RSV*_ (18%) with moderate SE% (43%). VPC (**Figure** 3) and GOF plots (**Figures S6, S12–14**) show appropriate description of RSV data and associated variability.

### Comparison of *in vitro* and *in vivo* rifampicin OATP Ki

**Figure 5:**
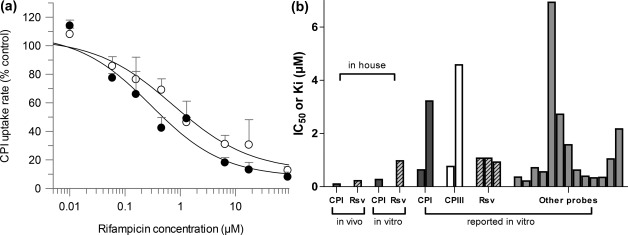
**(a)** Concentration‐dependent inhibition of CPI OATP‐mediated uptake in human hepatocytes using rifampicin, following preincubation with a buffer (○) or inhibitor (●) for 60 min. **(b)** Comparison of rifampicin model‐based *in vivo* Ki estimates with *in vitro* IC_50_/Ki estimates obtained in human hepatocytes (current study) or reported in OATP1B1 transfected cell line using different probes. Details of literature reported *in vitro* studies listed in **Table S5**.

RIF inhibited CPI and RSV uptake in human hepatocytes in a concentration‐dependent manner (**Supplementary Material**). Preincubation with RIF increased the potency of inhibition of OATP1B‐mediated CPI uptake by ∼2‐fold and resulted in IC_50_ of 0.30 μM (**Figure** 5 **a**). In contrast, no preincubation effect on RIF potency was observed using RSV as a probe in the same donor (IC_50_ = 1 μM, **Figure S1**). Rifampicin *in vitro* data obtained in human hepatocytes following the preincubation step were 4‐fold higher than estimated *in vivo* Ki. In addition to hepatocyte data obtained in the current study, model‐based *in vivo* estimates were compared to *in vitro* inhibition parameters reported in OATP1B1‐transfected cell lines using 11 different probe substrates (**Table S5**). Overall, *in vivo* Ki estimates were lower compared to the *in vitro* data; in some cases the difference was up to 50‐fold depending on the *in vitro* probe used.

### Power calculations

**Figure 6:**
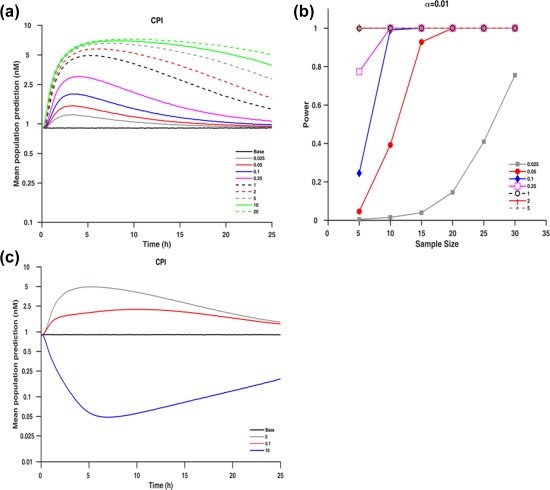
**(a)** Median simulated plasma concentration of CPI at different I/Ki ratios relative to rifampicin. **(b)** Power curves at significance levels (α = 0.01) for the different hypothetical I/Ki ratios based on a one‐sample paired *t*‐test of the ratio of logarithmic transformed AUC. **(c)** Median simulated plasma concentration of CPI assuming rifampicin inhibition of *k*_*syn*_ using hypothetical ratios of 0, 0.1, and 10 relative to the effect of rifampicin on *CL*_*b,CPI*_ where ratio of 0 corresponds to scenario of no inhibition of ksyn (transporter inhibition only).

The analysis above, together with marginal interindividual variability in CPI, confirms its utility as an endogenous biomarker for the identification of potent OATP1B inhibitors. Due to a current lack of CPI clinical data with a panel of inhibitors, the CPI model developed was used to simulate median CPI plasma concentration profiles for different hypothetical inhibitors (**Figure** 6 **a**). The simulated hypothetical inhibitors varied in their potency or exposure relative to RIF I/Ki (expressed as 0.025–20 ratio). The mean simulated CPI AUCR were 1.15, 1.26, 1.48, 2.02, 3.46, 4.37, 5.55, 6.31, and 6.9 for hypothetical inhibitors with I/Ki ratios of 0.025, 0.05, 0.1, 0.25, 1, 2, 5, 10, and 20 relative to the RIF scenario, respectively. The power to detect a significant DDI was dependent on the sample size, I/Ki ratio, and significance level set. The current analysis showed the ability to identify a moderate interaction (predicted AUCR >2) for scenarios with I/Ki ≥0.25 relative to RIF. Use of criterion of α = 0.01 and power of 0.8 required a sample size of 10 subjects. To detect a weak but clinically relevant OATP1B DDI using CPI as a probe (AUCR >1.25 cutoff, simulated I/Ki ≥0.05 relative to RIF) a minimum of 15 subjects with functional *SLCO1B1* are required (α = 0.01, **Figure** 6 **b**). Power analysis based on α = 0.05 is shown in **Figure S15**.

## DISCUSSION

In recent years efforts have been made to identify suitable endogenous biomarkers for transporter‐mediated DDIs.^4^, ^7^ However, knowledge gaps still exist in terms of understanding synthesis rate and elimination processes of biomarkers, verifying their specificity and utility to support/guide DDI study design. In the current study, plasma and urine CPI data ± potent OATP inhibitor RIF were utilized to develop a semimechanistic CPI model. Biliary excretion was the dominant CPI elimination route, with a maximal contribution of >85% under basal conditions. This analysis emphasizes the importance of informative clinical data when evaluating endogenous biomarkers; in this case, availability of both plasma and urine data (in baseline and RIF condition) was crucial for the identifiability of certain model parameters (*V*_*CPI*_).

In addition, a modeling approach was used to estimate the *in vivo* RIF OATP Ki using CPI data, assuming that OATP1B1/3‐mediated hepatic uptake is the rate‐determining step in CPI hepatic clearance. In the CPI model, RIF only impacts its biliary clearance (no effect on CPI renal clearance), which is supported by available urine data in both conditions^6^ and *in vitro* studies.^14^ Consequently, the Ki obtained by modeling of clinical data reflects RIF inhibition of OATP1B‐mediated transport of CPI. It is important to note that recent studies suggest that CPI is also a substrate for MRP2 and its transport is inhibited by RIF, with an IC_50_ of 83 μM.^16^ Considering a previously reported RIF unbound liver‐to‐plasma concentration ratio of 3.3 in humans^8^ and that maximum unbound plasma concentration observed here was >25 times lower than the MRP2 IC_50_, the impact of this interaction on CPI systemic exposure is expected to be negligible. Any potential MRP2 inhibition is anticipated to affect CPI liver exposure, analogous to previous analysis of the interplay of different hepatic processes on the liver AUC.^17^, ^18^, ^19^

Similar to CPI, estimation of RIF *in vivo* OATP Ki using plasma and urine data was obtained for RSV assuming that OATP inhibition is the driving force for increased plasma exposure. An ∼2‐fold difference between the unbound *in vivo* RIF Ki estimated using CPI and RSV (0.13 vs. 0.25 μM, respectively) could be attributed to differences in study design (steady‐state of the endogenous probe vs. single RSV dose), selectivity of probes, and differential contribution of OATPs to their hepatic uptake. CPI shows high affinity for OATP1B1 and OATP1B3, with no in involvement of OATP2B1‐mediated uptake,^11^ whereas RSV is transported by OATP2B1, NTCP, and BCRP,^20^, ^21^, ^22^ in addition to OATP1B1/1B3. Considering the weak inhibitory potency of RIF against NTCP and BCRP,^21^ the interaction with these transporters is not anticipated to contribute to more pronounced changes in RSV systemic exposure. Data on the potency of RIF OATP2B1 inhibition are inconsistent (IC_50_ 2.1–81 μM),^21^, ^23^ and hence inhibition of this hepatic transporter may be an additional contributing factor to the wider range of RSV AUCR observed. All the above indicate high selectivity of CPI for OATP1B and that the estimated fT values are likely to be attributed solely to OATP1B1/1B3, in contrast to RSV.

The uncertainty in the literature reported *in vitro* inhibition parameters (30‐fold range in RIF IC_50_, **Table S5**) and substrate‐dependent inhibition evident for OATP1B1^24^, ^25^ makes the translational aspect of OATP1B DDIs challenging, even for prototypical inhibitors like RIF. In addition, methodological considerations, e.g., preincubation with an inhibitor, have been reported to increase the potency of OATP1B inhibition *in vitro*,^26^, ^27^ as recently acknowledged by the US Food and Drug Administration (FDA) DDI guidance.^28^ RIF model‐based *in vivo* Ki values were 3–4‐fold lower compared with *in vitro* parameters obtained in human hepatocytes with the corresponding probes and following preincubation with inhibitor (**Figure** 5 **b**). None of the previous literature RIF inhibition studies considered the preincubation effect, with the exception of the current analysis and recent study.^29^ Although the shift in RIF inhibition potency with preincubation was not as prominent as reported in the case of cyclosporine,^26^ it reduced the disconnect between *in vitro* and *in vivo* inhibition parameter estimates.

The availability of interaction data for the biomarker and clinical probe in the same subjects provided an excellent opportunity to directly validate the utility of CPI as a biomarker for OATP1B‐mediated DDIs. Low intra‐ and interindividual variability was essential in defining CPI synthesis and estimating corresponding clearance parameters. No effect of circadian rhythm was evident on CPI baseline (**Figure** 3), in contrast to some of the other promising OATP1B endogenous biomarkers,^4^ where high variability in the baseline levels raises concerns on the ability of a biomarker to identify correctly DDI risk with moderate/weak inhibitors. Additionally, several of the proposed OATP1B endogenous biomarkers (e.g., hexadecanedioate) are also eliminated by renal transporters,^12^, ^30^ making it difficult to investigate DDI risk at a local tissue level. Metabolic stability of CPI was a key advantage in the current modeling exercise to estimate RIF Ki; endogenous biomarkers that undergo metabolism such as bile acids would require a more complex model structure to account for the synthesis rate, formation of metabolites, and potential recirculation, as done recently for bilirubin.^31^ The potential effect of a transporter inhibitor on the biosynthesis of a biomarker is an additional consideration in the biomarker qualification process. Simulations in **Figure** 6 **c** clearly illustrate potential bias in the interpretation of the biomarker DDI data if synthesis rate was also affected by transporter inhibitor investigated. Rapid recovery of CPI is an important consideration in the clinical study design, as CPI DDI profiles are expected to closely follow the perpetrator concentration–time profile, as illustrated nicely in RIF case (I/Ki on average >10 up to 8 h after RIF administration).

The existing clinical data and modeling confirmed validity of CPI for the investigation of OATP1B‐mediated DDIs with potent inhibitors such as RIF. As a likely comedication, RSV has been used to evaluate DDI risk with weak and moderate OATP1B inhibitors (**Table S6**), but direct comparison to CPI and its ability/sensitivity to identify DDIs with those inhibitors is not possible due to lack of data. To address this data gap, model‐based simulations were performed to predict changes in CPI exposure for hypothetical moderate and weak inhibitors with corresponding power calculations to guide optimal design of those clinical DDI studies. The power analysis showed that relatively small sample sizes are needed to clinically detect the effect of moderate and weak OATP1B inhibitors and corresponding changes in CPI plasma exposure. It is important to note that simulations were informed and based on the RIF PK model and existing data. Additional clinical studies with a panel of OATP1B inhibitors are required for further qualification of the CPI model developed. In addition, the CPI model is based on the data obtained in subjects with wildtype *SLCO1B1* c.521 T>C genotype, and therefore the impact of this clinically relevant polymorphism as a covariate on either baseline levels or DDI potential could not be considered in the model development.

In conclusion, the current study confirms the sensitivity of CPI to identify weak to potent OATP1B inhibitors in an adequately powered clinical study and highlights the general challenges associated with modeling of clinical data for endogenous biomarkers, in particular if their intended purpose is to support the DDI assessment in early stages. Availability of CPI and RSV interaction data in the same subjects showed no direct correlation in the DDI magnitude between the probes, most likely due to the promiscuous nature of RSV as an OATP1B probe; whether correlation could be established with a more selective OATP1B substrate (e.g., pitavastatin) remains to be seen. Further refinement of the CPI model towards a physiologically based PK model requires *in vitro* transporter kinetic data for CPI together with additional pharmacogenomic and clinical DDI studies with a panel of inhibitors. This would enable stepwise model qualification and simulations of systemic and liver CPI exposure under different scenarios (e.g., DDI risk for subjects with OATP1B1*5).

## METHODS

### Clinical data

Individual plasma and urine data were obtained from a previous study in 12 healthy male subjects.^6^ The study was split into three occasions (OCC1–3) with a 7‐day washout period between OCC1 and 2 and OCC2 and 3. During each occasion, baseline CPI urine and plasma samples were collected (**Figure S2**). A single 600‐mg oral dose of RIF and a single 5‐mg oral dose of RSV were administered to the volunteers in OCC1 and OCC2, respectively. Finally, in OCC3 the same respective doses of RIF and RSV were coadministered to healthy volunteers. Plasma (RIF, RSV, and CPI) were collected at predefined timepoints over the 24‐h period following administration of RIF and/or RSV. Urine samples were collected over one time interval (−7 to 0 h) for the pretreatment sample (CPI monitored) and two subsequent intervals (0–7 h and 7–24 h) for posttreatment samples of CPI and RSV. The total number of plasma samples for modeling purposes were 276 (OCC1 = 144, OCC3 = 132), 420 (OCC1 = 144, OCC2 = 144, OCC3 = 132), and 264 (OCC2 = 132, OCC3 = 132) for RIF, CPI, and RSV, respectively, whereas the total number of urine samples were 102 (pretreatment = 34, posttreatment = 68) and 44 for CPI and RSV, respectively. No subjects enrolled in the clinical study had OATP1B1*5 or OATP1B1*15 mutations, associated with altered OATP1B1 activity.

Variability in baseline CPI plasma concentration between the three occasions was assessed and a one‐way analysis of variance (ANOVA) test was used to determine any significant differences. CPI and RSV plasma concentrations in the presence/absence of RIF were used to calculate the area under the curve (AUC) using the trapezoid function in MATLAB (MathWorks, Natick, MA, 2015a). Subsequently, the fold change in RSV and CPI AUC of each individual and the fraction eliminated by transporters (fT) for each probe was calculated (Equation 1).

(1) $$fT=1-\frac{AUC\left(control\right)}{AUC\left(+inhibitor\right)}$$

### Structural and statistic models used for population PK analysis

Population PK models were developed sequentially for analysis of RIF, CPI, and RSV plasma and urine data using nonlinear mixed‐effects modeling software, NONMEM, with first‐order conditional estimation method.^32^ Statistical analysis, GOF plots, and other graphical analysis were conducted in MATLAB. The structural models for RIF and RSV (**Supplementary Material**) were selected based on population PK models available in the literature.^34^, ^35^ An exponential model was used to describe interindividual variability random‐effects parameters and IOV was evaluated for the model parameters; occasions were defined as periods of different visits following recruitment or washout periods. Covariates and random‐effects parameters (IIV and IOV) were retained in the models when there was a significant change in objective function and improvement in GOF plots. The incorporation of IIV and IOV in the models is shown in Equation 2:

(2) $$\theta_{ik}=\theta \cdot e^{\eta_{i}+\kappa_{ik}}$$

where *θ*_*ik*_ is the parameter for i^th^ individual on k^th^ occasion, *θ* is the population (typical individual) parameter, *η*_*i*_ is the parameter that describes the deviation of the i^th^ individual parameter from the population (IIV), and *k*_*ik*_ the parameter that describes the deviation of the i^th^ individual from the population in addition to *η*_*ik*_ at the k^th^ occasion (IOV). Both random effect parameters (*η*_*ik*_ and *k*_*ik*_) are assumed to be randomly distributed with a mean of zero and the variances are estimated during analysis. Proportional or additive or combination of proportional and additive residual error models were investigated for the residuals.

### CPI population PK model

The plasma concentrations of the endogenous biomarker CPI were described using a turnover model (Equation 3).^36^ Simultaneous fitting of individual CPI plasma and urine data in the presence and absence of RIF was performed. In the final model, RIF inhibited biliary elimination (*CL*_*b,CPI*_) of CPI, whereas its renal clearance (*CL*_*R,CPI*_) and *k*_*syn*_ were not affected. The rate of change of CPI plasma concentration under baseline and RIF condition is shown in Equations 3 and 4, respectively, whereas change in CPI amount in urine over time is shown in Equation 5.

(3) $$\frac{dC_{CPI}}{dt}=\left[k_{syn}-CL_{b,CPI}\cdot C_{CPI}-CL_{R,CPI}\cdot C_{CPI}\right]\cdot \frac{1}{V_{CPI}}$$

(4) $$\frac{dC_{CPI}}{dt}=\left[k_{syn}-\frac{CL_{b,CPI}\cdot C_{CPI}}{1+\frac{C_{RIF}}{Ki_{CPI}}}-CL_{R,CPI}\cdot C_{CPI}\right]\cdot \frac{1}{V_{CPI}}$$

(5) $$\frac{dU_{CPI}}{dt}=CL_{R,CPI}\cdot C_{CPI}$$

where *C*_*CPI*_ is its plasma concentration, *U*_*CPI*_ is the CPI amount in urine, t is time, *k*_*syn*_ is the zero‐order synthesis rate, *Ki*_*CPI*_ is RIF transporter inhibition constant using CPI as a probe, *V*_*CPI*_ is the volume of distribution of CPI and *C*_*RIF*_ is RIF plasma concentration (linked to RIF model). Simulations were also performed to assess the consequences of the hypothetical inhibition of both CPI synthesis (*k*_*syn*_ and biliary elimination (*CL*_*b,CPI*_) by inhibitor of interest (details in **Supplementary Material**).

Formal structural identifiability analysis of this model was conducted in DAISY^37^ to determine whether the model and the parameter estimates are identifiable globally or locally^38^ using the available CPI plasma and urine data ± RIF. The availability of such data is particularly important because of the endogenous nature of CPI, which may affect identifiability of *V*_*CPI*_ from the plasma data in isolation. An analogous population PK approach was undertaken for RIF and RSV (details in **Supplementary Material**).

### Power calculations

To assess the utility of CPI to identify weak and moderate OATP1B inhibitors, the RIF and CPI models were used to simulate CPI plasma concentrations using adjusted C_RIF_/Ki_(CPI)_ ratios in Equation 4, illustrating either a different inhibitor potency or plasma concentration/dose level, over several order of magnitude (0.025–20) relative to RIF. Parameter estimates including random effect parameters obtained from the analysis were used for the simulations. Plasma concentrations were simulated in MATLAB and area under the plasma concentration–time curve (AUC) was estimated using the trapezoidal rule with an in‐built function in MATLAB. The simulation was based on a two period one‐way crossover study design where in the first period plasma/urine samples of CPI are observed under baseline condition and following a period of washout the same subjects received a single dose of 600 mg RIF and plasma/urine samples of CPI were collected again for analysis. Power curves were calculated for each modified I/Ki ratio by performing a one‐sample paired *t*‐test on the ratio of logarithmic transformed AUC following simulation at predefined sample sizes.^5^, ^6^, ^7^, ^8^, ^9^, ^10^, ^11^, ^12^, ^13^, ^14^, ^15^, ^16^, ^17^, ^18^, ^19^, ^20^, ^21^, ^22^, ^23^, ^24^, ^25^, ^26^, ^27^, ^28^, ^29^, ^30^ At each sample size, the simulation and tests were repeated 5,000 times and the power of the test was calculated as the proportion of the simulations during which the null hypothesis was rejected at the significance level (0.05 and 0.01).

## CONFLICT OF INTEREST

The other authors declare no conflicts of interest.

## FUNDING

S.B. is supported by a PhD studentship from the Biotechnology and Biological Sciences Research Council, UK (BB/L502376/1) and UCB, UK.

## AUTHOR CONTRIBUTIONS

S.B., K.O., K.M., S.H., Y.L., W.G.H., and A.G. wrote the article; S.B., K.O., S.H., W.G.H., and A.G. designed the research; S.B. and K.O. performed the research; S.B. and K.O. analyzed the data.

## Supporting information

Supplementary Material
