# Supplementary material for: Gaining Mechanistic Insight Into Coproporphyrin I as Endogenous Biomarker for OATP1B‐Mediated Drug–Drug Interactions Using Population Pharmacokinetic Modeling and Simulation
Source: Clin Pharmacol Ther. 2018 Jan 17;104(3):564–74. doi: 10.1002/cpt.983 (PMC6175062; doi:10.1002/cpt.983)
Supplement: Supplementary file 1 — Supplementary Material [file CPT-104-564-s001.docx]

**Supplementary Material for the manuscript CPT 2017-0727 ‘Gaining mechanistic insight into coproporphyrin I as endogenous biomarker for OATP-mediated drug-drug interactions using population pharmacokinetic modelling and simulation’**

Shelby Barnett, Kayode Ogungbenro, Karelle Ménochet, Hong Shen, Yurong Lai, W. Griffith Humphreys and Aleksandra Galetin

**Determination of RIF IC_50_ in plated human hepatocytes using CPI and rosuvastatin as probes**

**Methods**

The *in vitro* IC_50_ for rifampicin was assessed using CPI and RSV as OATP probe substrates in plated human hepatocytes. Cryopreserved human hepatocytes (Donor QQE, BioreclamationIVT, Baltimore, MD) were thawed and plated at a density of 350,000 cells ([1](#_ENREF_1)). After culturing for 4h, hepatocytes were washed twice with Dulbecco's phosphate-buffered saline and pre-incubated for an hour with either buffer or rifampicin (0.01-100µM). Following pre-incubation, the medium was replaced with CPI (0.30µM) or RSV (2µM) containing rifampicin (0-100µM). Incubations were stopped after 3 or 5 minutes (for RSV and CPI,respectively) using the washing protocol described previously ([2](#_ENREF_2)). Monolayers were lysed with water and frozen overnight and then prepared for protein quantification (BCA assay) and LC-MS/MS analysis. Experimental conditions used, i.e., low probe substrate concentrations (<<Km) ensured unbiased parameter estimates regardless of the transporter inhibition mechanism and hence IC_50_ = Ki.

**LC-MS/MS analysis of CPI and RIF *in vitro* inhibition data in human hepatocytes**

Lysate samples were prepared in 50% internal standard (IS) (0.5µM Glibenclamide). CPI samples were run on a Shimadzu Nexera X2 LC system attached to Sciex 5500 QTRAP using a Phenomenex C18 50 x 2.1 mm, 5µM column at a flow rate of 0.8mL/ min using the solvent gradient described in Table S1 alongside the mass spectrometry conditions highlighted in Table S2.

**LC-MS/MS analysis of RSV and RIF *in vitro* inhibition data in human hepatocytes**

Lysate samples were prepared in 50% internal standard (IS) (0.1µM Naloxone). RSV samples were run on a Quattro Ultima (Waters) with Agilent 1100 HPLC using a Phenomenex Luna C18 50x4.6mm 3µM column at a flow rate of 1mL / min using the solvent gradient described in Table S3 alongside the mass spectrometry conditions highlighted in Table S4. The lower limit of quantification (LLOQ) was 2.5nM and 0.2nM for RSV and CPI, respectively. The lowest concentration of the probe measured (in the presence of the highest RIF concentration) was >3-fold above the LLOQ for the corresponding drug.

**Table S1**: Solvent gradient for LC analysis of CPI and RIF

| A: Water (0.1% Formic acid) | | |
| --- | --- | --- |
| B: Acetonitrile (0.1% Formic acid) | | |
| Time (min) | % A | % B |
| 0.01 | 95 | 5 |
| 0.02 | 95 | 5 |
| 2.2 | 5 | 95 |
| 2.6 | 5 | 95 |
| 2.65 | 95 | 5 |
| 3.49 | 95 | 5 |
| 3.5 | System Controller Stop | |

**Table S2**: LC-MS/MS conditions for analysis of CPI and RIF in lysate samples

| Compound | Q1 Mass (Da) | Q3 Mass (Da) | Dwell (msec) | DP (volts) | EP (volts) | CE (volts) | CXP (volts) |
| --- | --- | --- | --- | --- | --- | --- | --- |
| CPI | 655.284 | 537 | 50 | 11 | 10 | 77 | 0 |
|  |  | 464.2 | 50 | 11 | 10 | 87 | 16 |
| Rifampicin | 823.403 | 791.4 | 50 | 16 | 10 | 23 | 4 |
|  |  | 95 | 50 | 16 | 10 | 99 | 18 |
| IS | 494.141 | 169 | 50 | 51 | 10 | 45 | 8 |

**Table S3**: Solvent gradient for LC analysis of RSV and RIF

| A = 90% H2O, 10% MeOH + 0.05% formic acid  B = 10% H2O, 90% MeOH + 0.05% formic acid  C = 90% H2O, 10% MeOH + 1mM ammonium acetate  D = 10% H2O, 90% MeOH + 1mM ammonium acetate | | | | |
| --- | --- | --- | --- | --- |
| Time (min) | % A | % B | %C | %D |
| 0 | - | - | 100 | - |
| 1 | - | - | 100 | - |
| 1.1 | - | 5 | 20 | 75 |
| 3 | - | 5 | 20 | 75 |
| 3.1 | - | - | - | 100 |
| 5 | - | - | - | 100 |
| 5.1 | - | - | 100 | - |
| 6.5 | - | - | 100 | - |

**Table S4**: LC-MS/MS conditions for analysis of RSV in lysate samples

| Compound | Q1 Mass (Da) | Q3 Mass (Da) | Cone Voltage (V) | Collision gas (eV) |
| --- | --- | --- | --- | --- |
| RSV | 482.8 | 259.0 | 90 | 35 |
| IS | 328.85 | 310.90 | 90 | 20 |

**Estimation of RIF IC_50_ in plated human hepatocytes**

Lysate concentrations were used to demine uptake rates over a series of free RIF concentrations measured in the medium and plotted as a % of the control to estimate RIF IC_50_ (Main article Figure 5A and Figure S1). RIF IC_50_ was determined using equation 1, where U denotes the uptake rate (% vs control) at the free inhibitor concentration in the incubation [I] (µM); R, the range of inhibition; B, the un-inhibitable background (%), and S, the slope factor (GraFit v6).

Model estimated RIF *in vivo* Ki values were compared to inhibition data generated in the current study in human hepatocytes. In addition, literature reported *in vitro* RIF inhibition data in OATP1B1-transfected cell lines using either CPI or other OATP1B1 probes (Table S5) were collated to understand further any potential differences between *in vitro* and *in vivo* estimated RIF inhibition parameters.

**
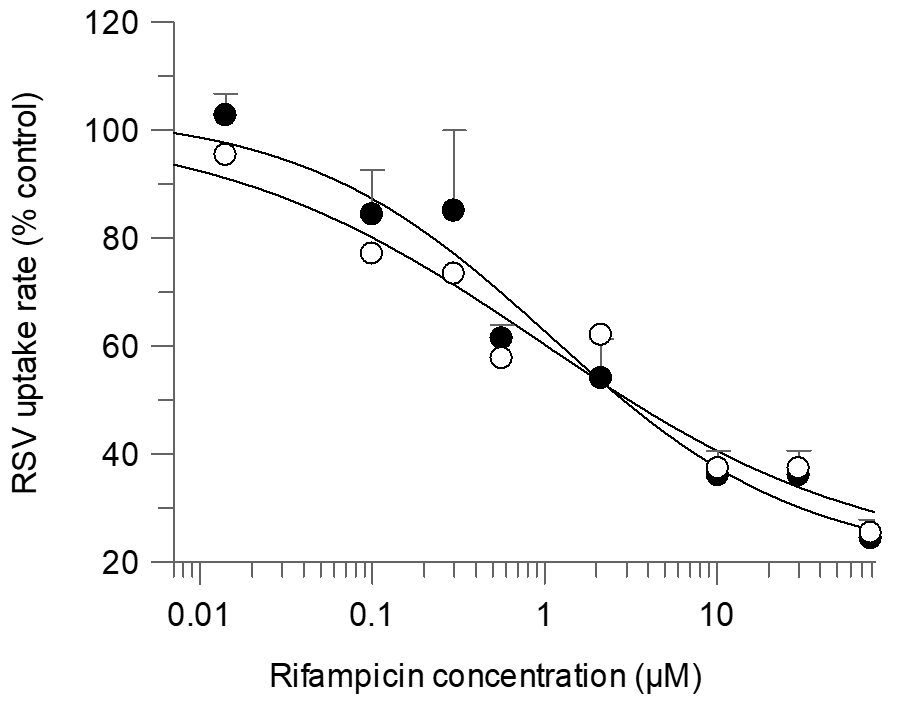
**$U=\frac{R}{1+\left(\frac{[I]}{\text{IC}_{50}}\right)^{s}}+\text{B}$ (1)

**Figure S1:** Concentration-dependent inhibition of rosuvastatin OATP-mediated uptake in human hepatocytes using rifampicin, following pre-incubation with a buffer (○) or inhibitor (●) for 60 minutes.

**Results**

Rifampicin showed concentration-dependent inhibition of CPI uptake in plated human hepatocytes resulting in an IC_50_ of 0.30µM (±0.15µM) and 0.73µM (±0.23µM) following pre-incubation with RIF and buffer, respectively. There was no effect of pre-incubation of RIF on the uptake of RSV IC_50_ = 1µM±0.5µM.These IC_50_ values were compared with those collated from the literature. IC_50_ values generated here using RSV as a probe are in line with the reported values in transfected OATP1B1 cells (Table S5). However, for CPI the IC_50_ values generated for RIF in this study are up to 10-fold lower than previously reported values using CPI as a substrate in transfected OATP1B1 cells.

**Table S5**: Collation of rifampicin *in vitro* Ki/IC_50_ values in transfected OATP1B1 cells

| **Substrate** | **Cell line** | **Substrate Concentration (µM)** | **Rifampicin Concentration (µM)** | **Pre-incubation with rifampicin** | **IC_50_ (µM)** | **Stdev** | **Reference** |
| --- | --- | --- | --- | --- | --- | --- | --- |
| Coproporphyrin I | CHO | 0.1 | 0.1-100 | 🗶 | 3.25 | 0.12 | ([3](#_ENREF_3)) |
| Coproporphyrin III | CHO | 0.1 | 0.1-100 | 🗶 | 4.61 | 0.14 | ([3](#_ENREF_3)) |
| Coproporphyrin I | HEK | 0.2 | 0.1-100 | 🗶 | 0.66 | 0.11 | ([4](#_ENREF_4)) |
| Coproporphyrin III | HEK | 0.2 | 0.1-100 | 🗶 | 0.79 | 0.16 | ([4](#_ENREF_4)) |
| GCDCA-S | HEK | 1 | 0.1-30 | 🗶 | 0.39 | 0.09 | ([5](#_ENREF_5)) |
| [^3^H]-E217bG | HEK | 1 | 0.01-25 | ✓ | 0.24 | 0.03 | ([6](#_ENREF_6)) |
| [^3^H]-E217bG | HEK | 1 | 0.01-25 | 🗶 | 0.74 | 0.08 | ([6](#_ENREF_6)) |
| [^3^H]-E_2_G | HEK | 0.1 | 0.01-100 | 🗶 | 0.585 | 0.074 | ([7](#_ENREF_7)) |
| [^3^H]-E_1_S | HEK | 0.01 | 0.01-100 | 🗶 | 6.96 | 1.31 | ([7](#_ENREF_7)) |
| BSP | HEK | 0.01 | 0.01-100 | 🗶 | 2.75 | 0.62 | ([7](#_ENREF_7)) |
| Rosuvastatin | MDCKII | 0.1 | 0.1-100 | 🗶 | 1.1 | 0.2 | ([8](#_ENREF_8)) |
| Rosuvastatin | HEK | 0.1 | - | 🗶 | 1.1 | 0.28 | ([9](#_ENREF_9)) |
| Rosuvastatin | HEK | 1 | 0.01-100 | 🗶 | 0.952 | 0.098 | ([10](#_ENREF_10)) |
| Pitavastatin | MDCKII | 0.1 | 0.1-100 | 🗶 | 1.6 | 0.1 | ([8](#_ENREF_8)) |
| Pravastatin | HEK | 10 | 0.01-100 | 🗶 | 0.653 | 0.117 | ([10](#_ENREF_10)) |
| Fexofenadine | HEK | 1 | 0.01-100 | 🗶 | 0.423 | 0.032 | ([10](#_ENREF_10)) |
| Nateglinide | HEK | 1 | 0.01-100 | 🗶 | 0.358 | 0.079 | ([10](#_ENREF_10)) |
| Valsartan | HEK | 1 | 0.01-100 | 🗶 | 0.377 | 0.022 | ([10](#_ENREF_10)) |
| Pitavastatin | HEK | 0.1 | 0.01-100 | 🗶 | 1.07 | 0.08 | ([10](#_ENREF_10)) |
| Atorvastatin | HEK | 1 | 0.1-630 | 🗶 | 2.2 | - | ([11](#_ENREF_11)) |

Rifampicin IC_50_ using CPI and CPIII as a substrate for OATP1B3 transfected cells are 0.25-1.61µM and 0.27-1.41µM, respectively ([3](#_ENREF_3), [4](#_ENREF_4))

**PK Modelling and Simulation Methods**

**Rifampicin population PK model**

Based on a previously developed population PK model for RIF, a one compartment first order elimination model with transit absorption model was used to describe plasma concentrations of RIF ([12](#_ENREF_12)). This model was parameterised using clearance (CL), volume of distribution (V), absorption rate constant (ka), mean transit time (MTT) and number of transit compartments (n). In order to account for the interaction between RIF and CPI and RIF and RSV, sequential analysis of plasma and urine data was conducted. A population PK model was developed for RIF plasma data only at the first stage; subsequently individual PK parameters for RIF were fixed to the empirical Bayes estimates for analysis of plasma and urine CPI and RSV data separately. A visual predictive check (VPC) of the final population PK model was conducted by simulation of 10,000 random individual profiles and the 95% prediction intervals of the simulations were superimposed with observed plasma or urine data. During the simulations, the mean urine volume at the time of collection in the observed data was used to convert model predicted concentration into amount.

### Rosuvastatin population PK model

Based on the available plasma and urine data, a two compartment first order absorption and elimination model was used to describe the PK of RSV in the subjects. The model was parameterised using $ka_{RSV}$, volume of distribution of central ($V1_{RSV}$) and peripheral compartments ($V2_{RSV}$), inter-compartmental clearance ($Q_{RSV}$), biliary ($CL_{b,RSV}$) and renal ($CL_{R,RSV}$) clearance parameters. Analogous to CPI, inhibition of $CL_{b,RSV}$ by RIF was incorporated in the rate of change of RSV plasma concentration, whereas it was assumed that RIF has no effect on RSV renal elimination (Eq 2). The rate of change of RSV amount in urine over time is shown in Eq3.

$\frac{dC_{RSV}}{dt}=\left[ka_{RSV}\cdot A_{D_{RSV}}-Q_{RSV}\cdot C_{RSV}+Q_{RSV}\cdot C_{P_{RSV}}-\frac{CL_{b,RSV}\cdot C_{RSV}}{1+\frac{C_{RIF}}{Ki_{RSV}}}-CL_{R,RSV}\cdot C_{RSV}\right]\cdot\frac{1}{V1_{RSV}}$ (2)

$\frac{dU_{RSV}}{dt}=CL_{R,RSV}\cdot C_{RSV}$ (3)

where $C_{RSV}$and $C_{P_{RSV}}$ are RSV concentrations in central (plasma) and peripheral compartments, $A_{D_{RSV}}$ is the amount in depot compartment, $U_{RSV}$ is RSV amount in the urine and $Ki_{RSV}$ is RIF transporter inhibition constant using RSV as a probe. Change in RSV distribution parameters ($Q_{RSV}$,$V1_{RSV}$ and $V2_{RSV}$) following co-administration with RIF was also investigated as a binary covariate where two sets of parameters were estimated under the two experimental conditions; RSV alone and RSV under treatment (presence of RIF).

### Simulation of the potential inhibition of both synthesis rate and biliary elimination of CPI

Currently, there are no data on the effect of RIF single dose on ALA synthase over 24h period ([13](#_ENREF_13)) and therefore no effect of RIF on CPI $k_{syn}$ was implemented in the model. Simulations were performed to assess the consequences of potential inhibition of both CPI synthesis ($k_{syn}$) and biliary elimination ($CL_{b,CPI}$) by inhibitor of interest using equation 4.

$\frac{dC_{CPI}}{dt}=\left[\frac{k_{syn}}{1+\left(\frac{C_{RIF}}{Ki_{CPI}}\right)^{'}}-\frac{CL_{b,CPI}\cdot C_{CPI}}{1+\frac{C_{RIF}}{Ki_{CPI}}}-CL_{R,CPI}\cdot C_{CPI}\right]\cdot\frac{1}{V_{CPI}}$ (4)

The effect on$k_{syn}$was investigated by simulating plasma concentration of CPI using (C_RIF_/Ki_(CPI)_)’ hypothetical relative ratios of 0, 0.1 and 10 of C_RIF_/Ki_(CPI)_, where ratio of 0 corresponds to scenario of no inhibition of $k_{syn}$ and inhibitory effect only on the transporter (reduces to equation 4 main article). Other scenarios simulated represent cases where either 10-fold lower or greater inhibition of$k_{syn}$is implemented relative to the effect of RIF on the transporter i.e., $CL_{b,CPI}$ in the model.


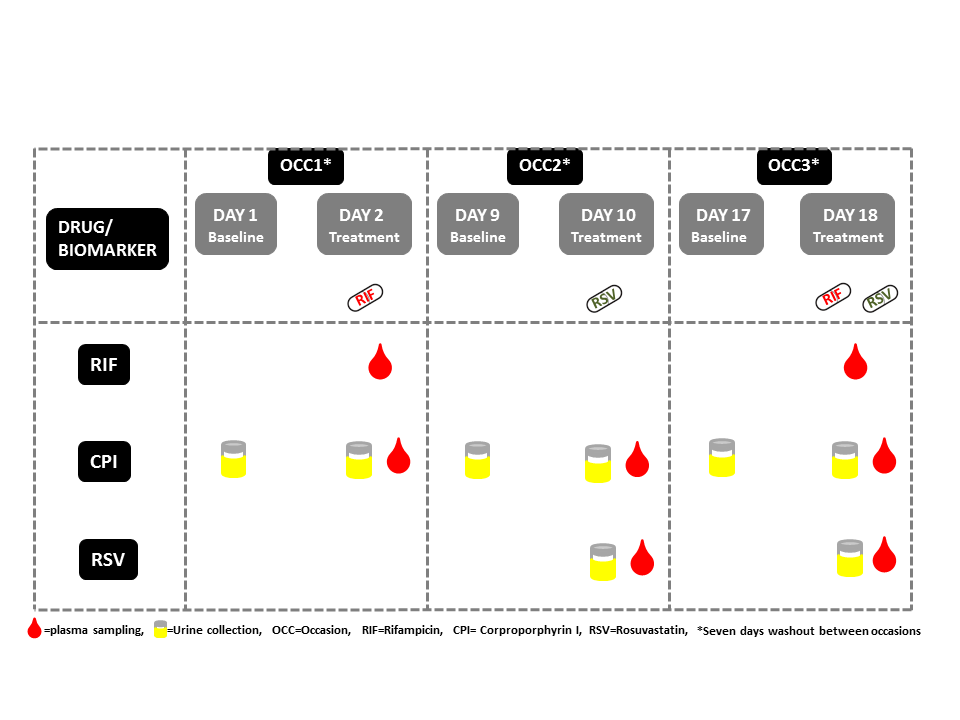
**Figure S2:** Schematic representation of experimental design of the clinical study


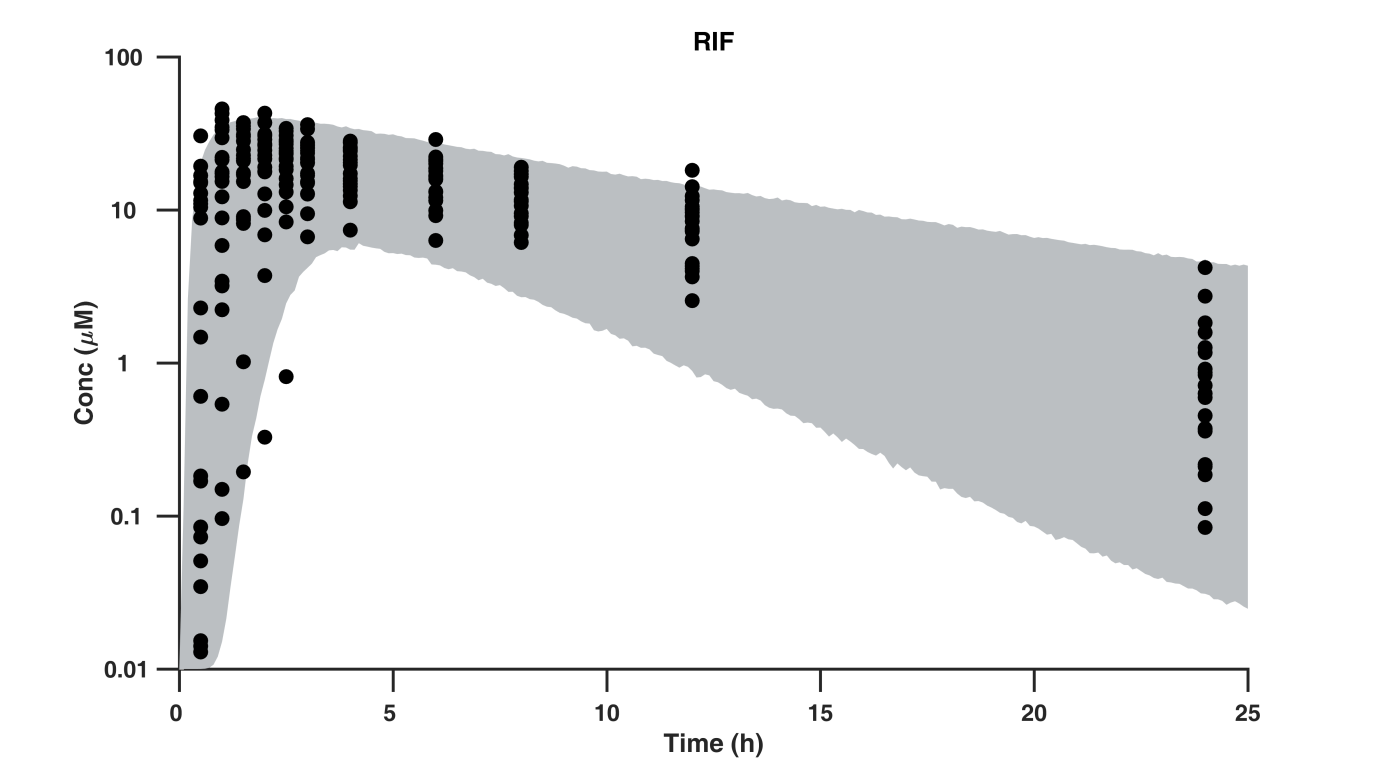


**Figure S3:** Visual predictive check of the developed population PK model for RIF plasma data, superimposed with the observed data. The grey area represents the 95% prediction of the simulated data and the dark circles are the observed data

| 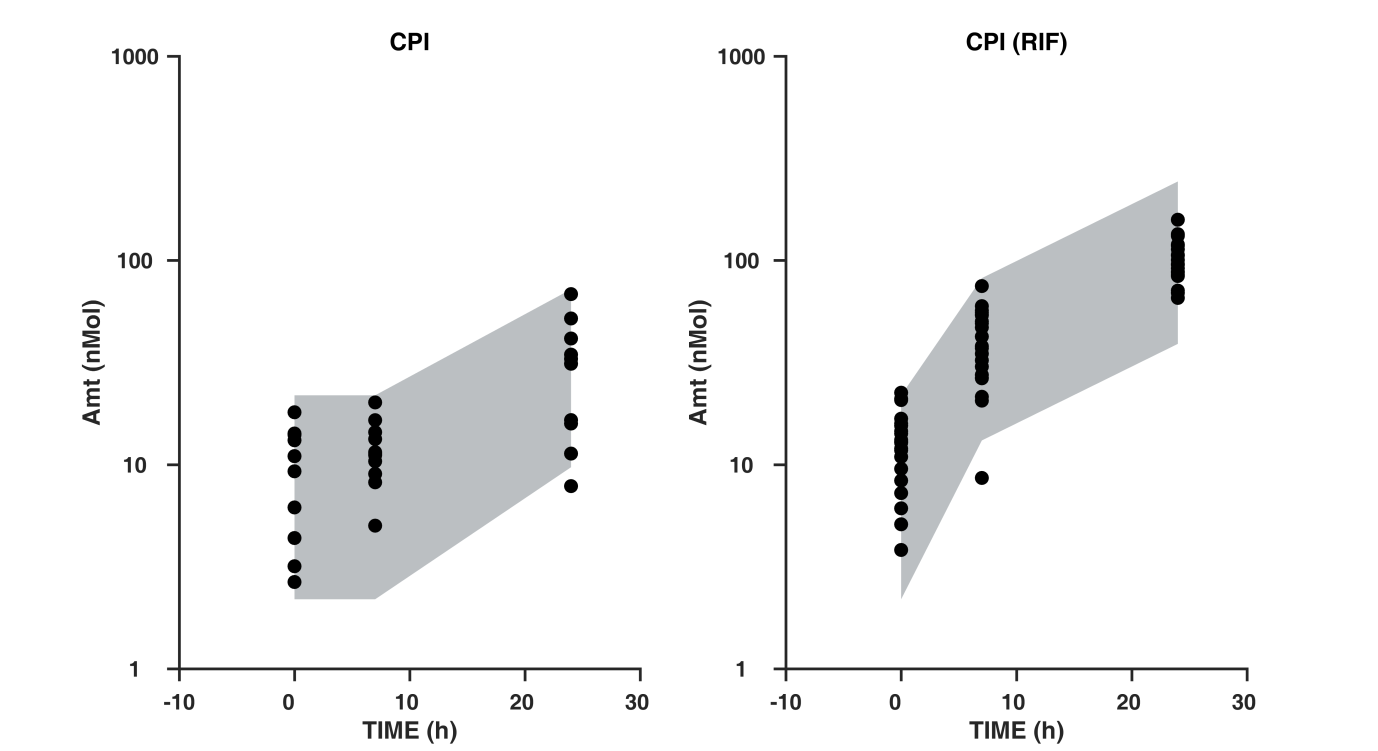 |
| --- |
| 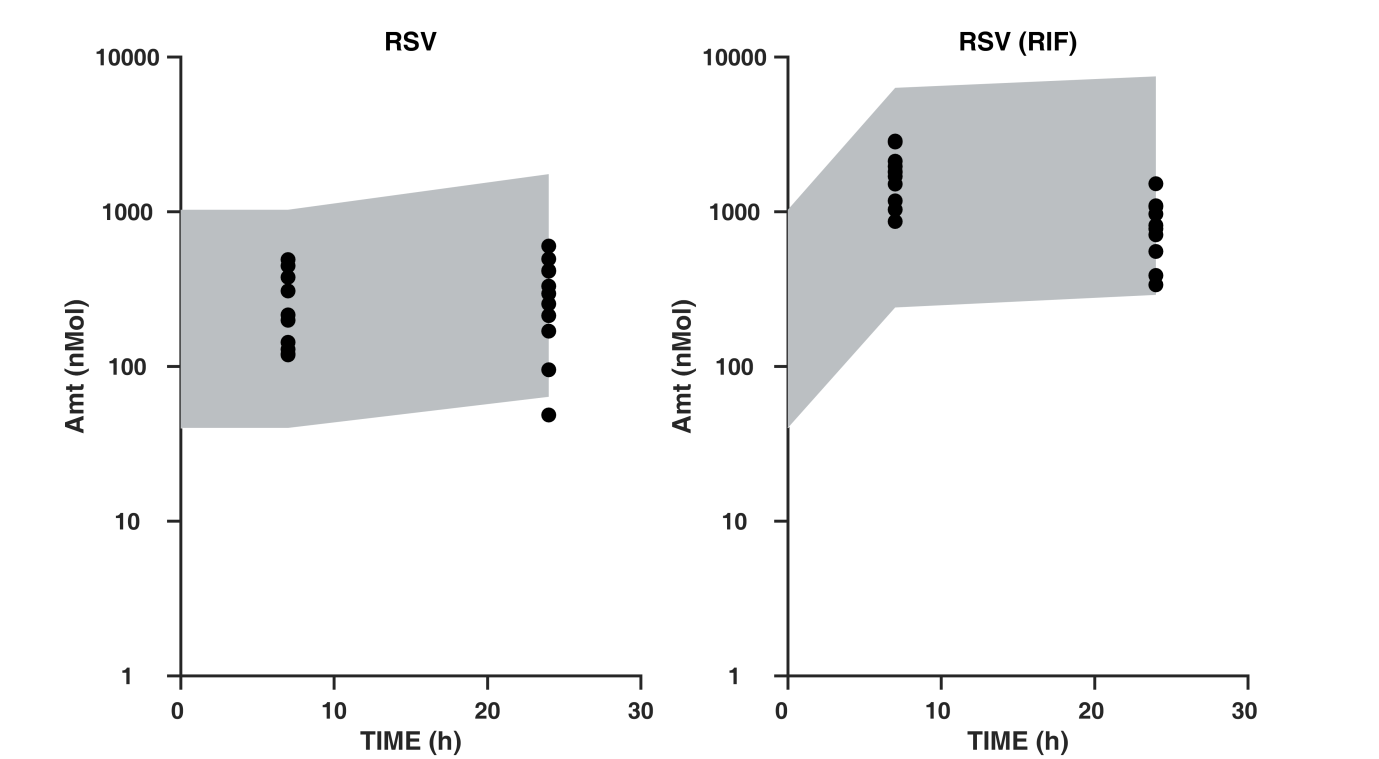 |

**Figure S4:** Visual predictive check (VPC) of the developed population PK model for CPI (Upper panel) and RSV (Lower panel) urine data, superimposed with the observed data. The grey area represents the 95% prediction of the simulated data and the dark circles are the observed data.

| 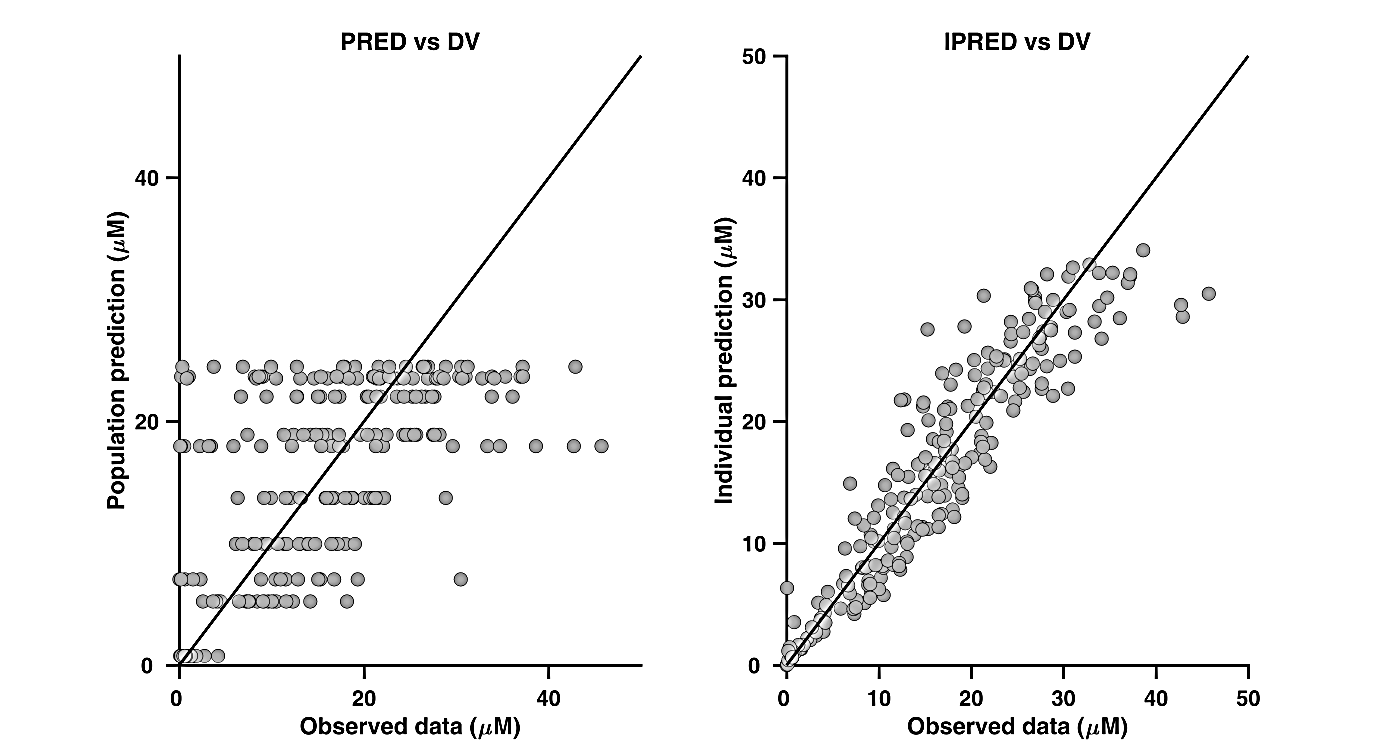 |
| --- |
|  |


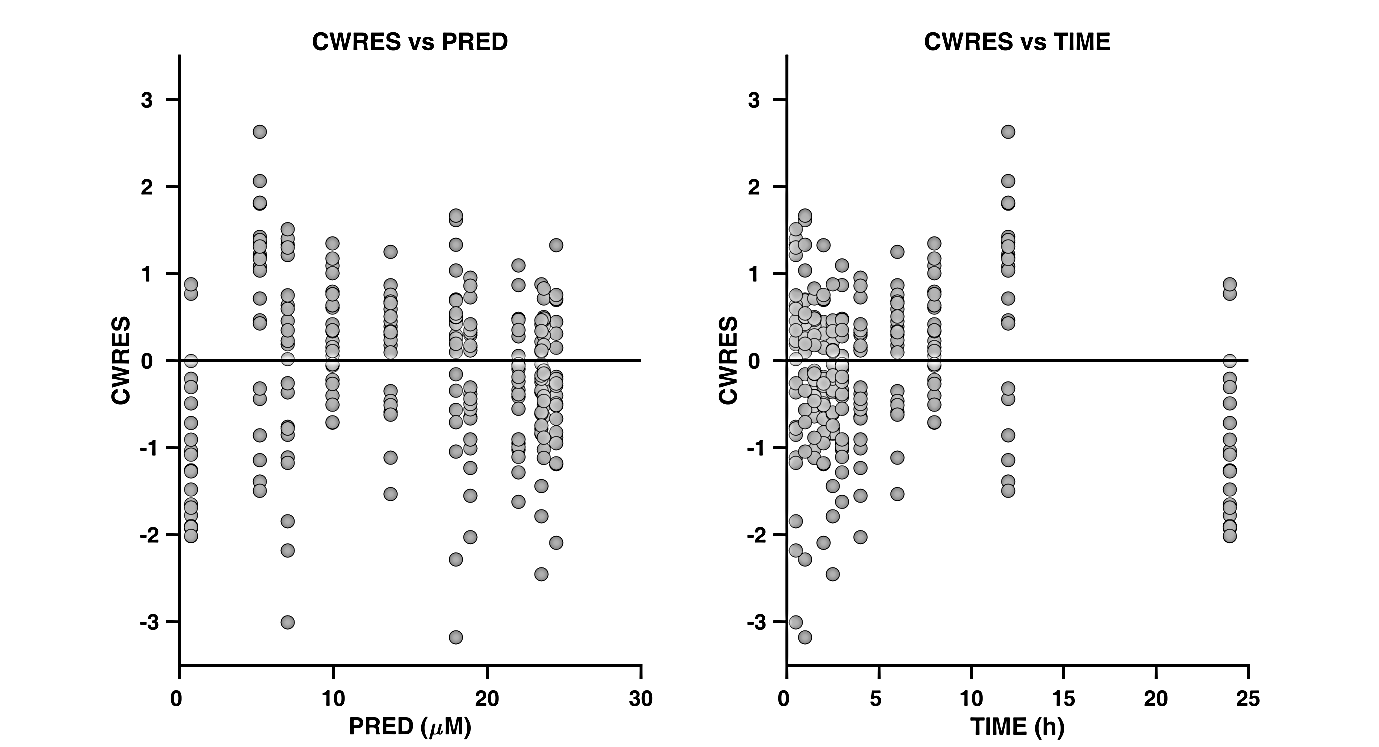


**Figure S5:** Goodness-of-fit plots for population PK model of RIF. DV, PRED, IPRED and CWRES are the observed data, population and individual model prediction and conditional weighted residuals respectively.

| **Plasma** | **Urine** |
| --- | --- |
| 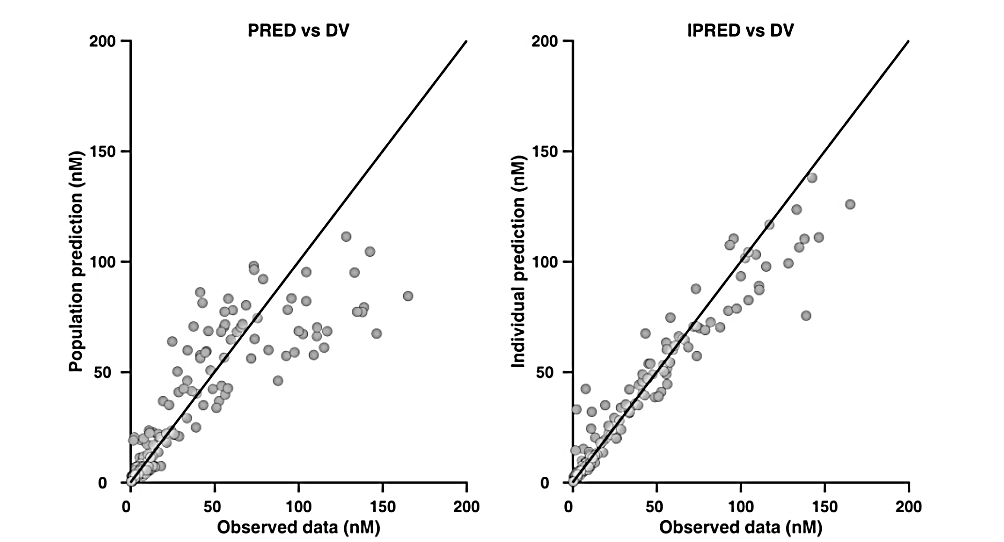 | 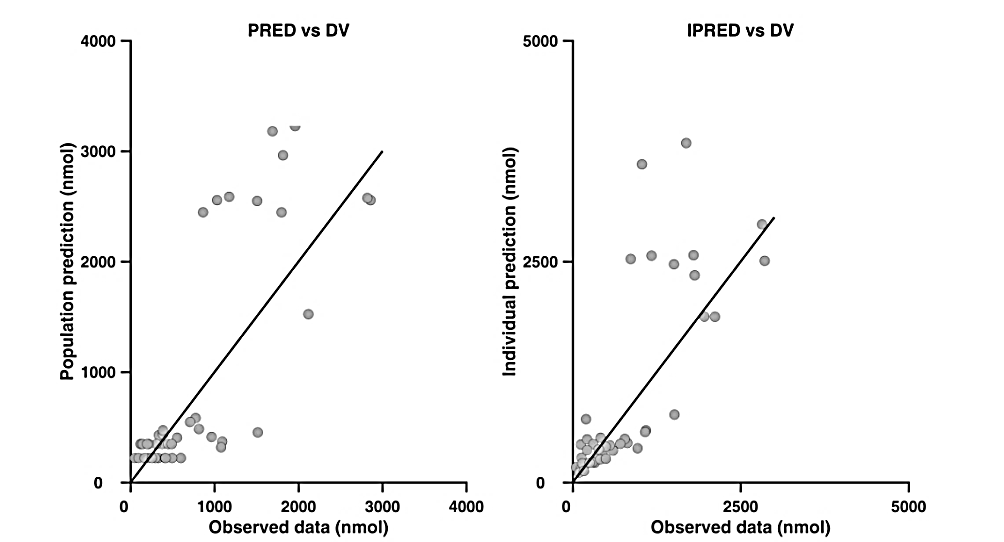 |
| 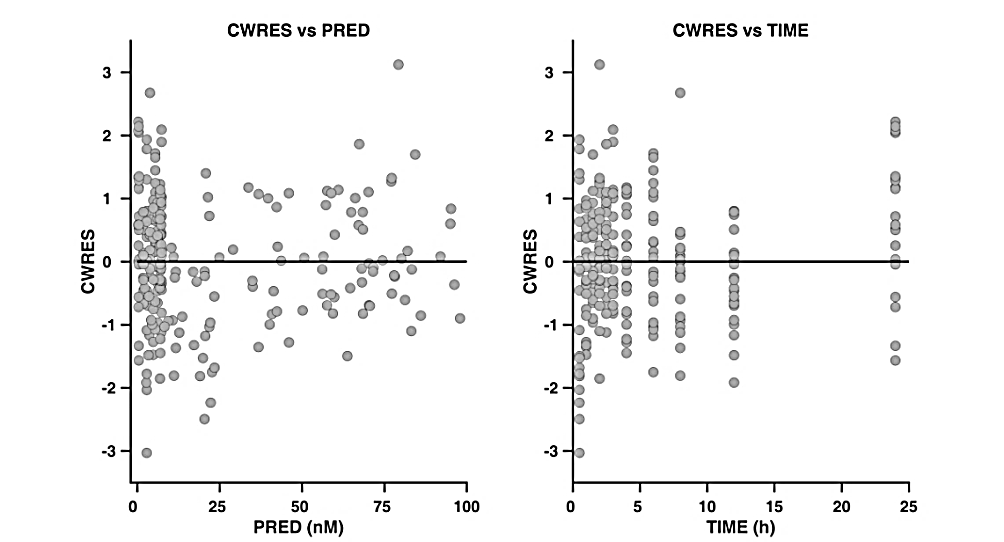 | 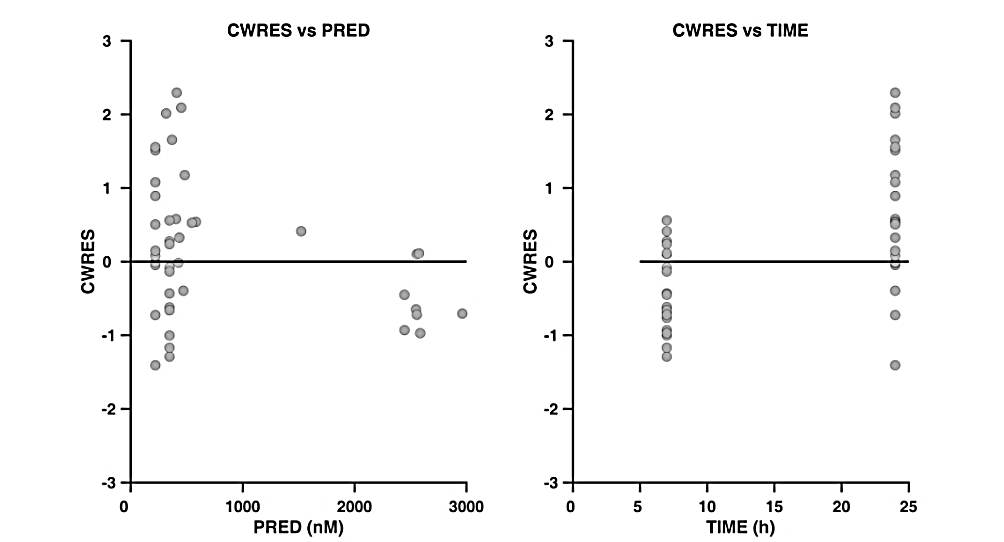 |

**Figure S6:** Goodness-of-fit plots for population PK model describing RSV plasma and urine data. DV, PRED, IPRED and CWRES are the observed data, population and individual model prediction and conditional weighted residuals respectively


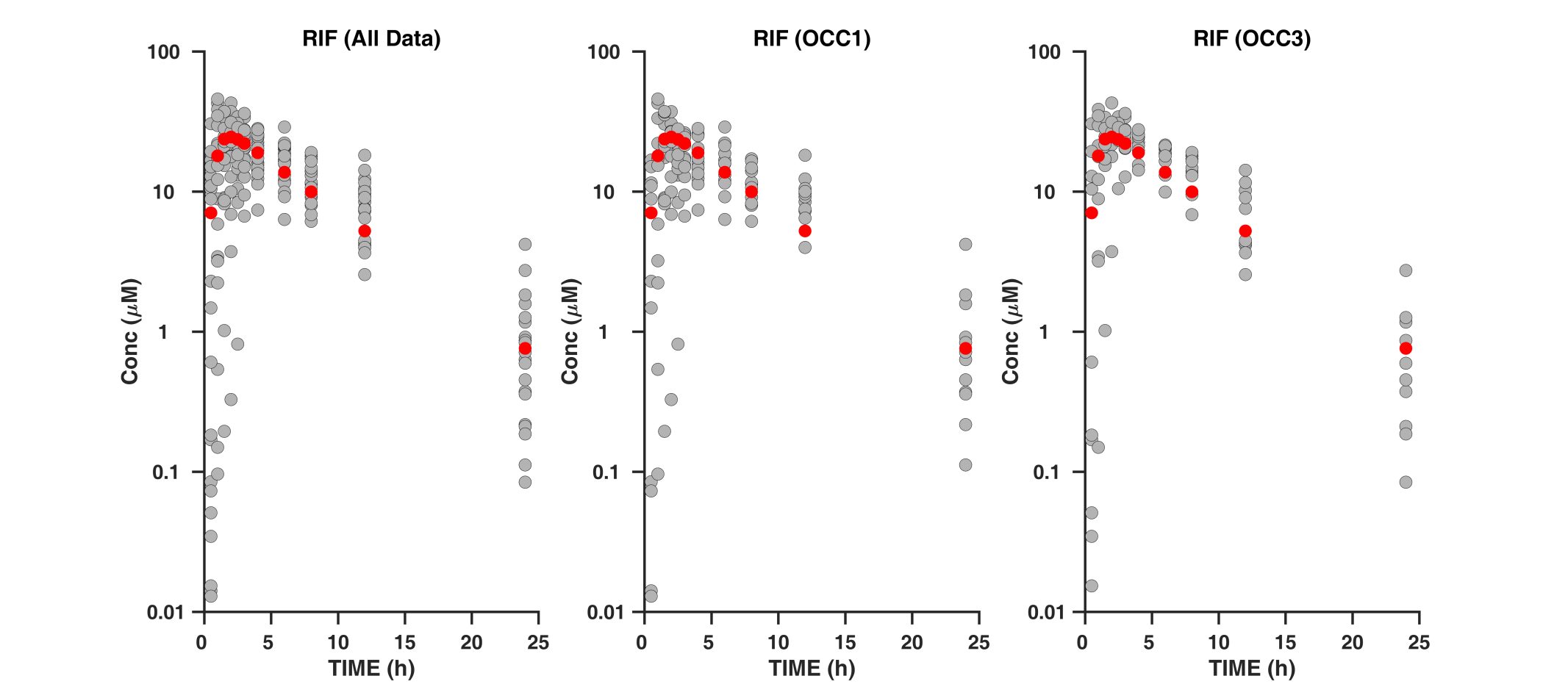


**Figure S7:** Observed plasma concentration data (grey circles) and the fitted population prediction (red circles) of RIF by the model for all data (All Data) and stratified by occasion (OCC1 and OCC3).

**
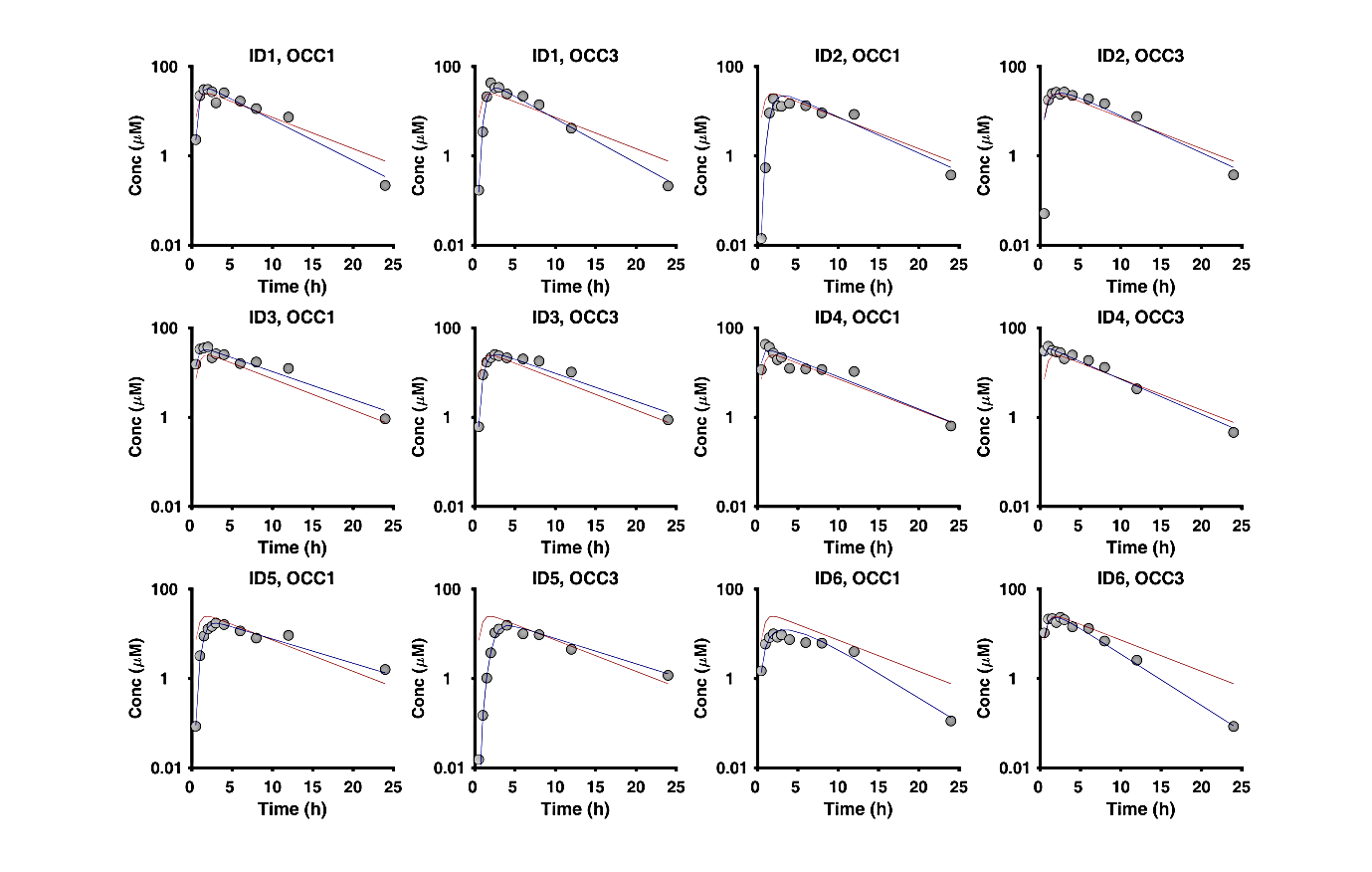

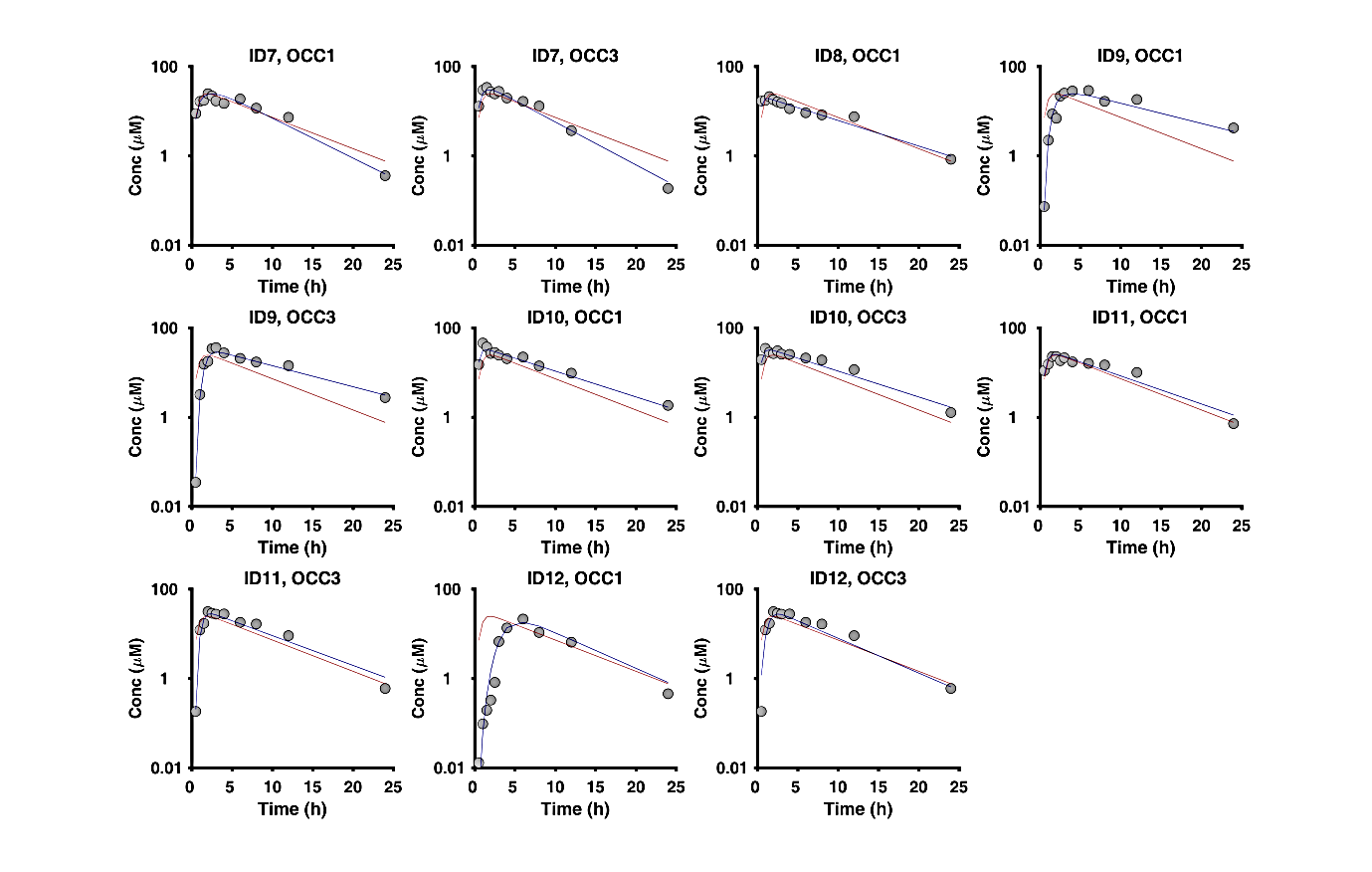
**

**Figure S8**: Individual plasma concentration data of RIF (grey circles) and the fitted population (red line) and individual prediction (blue line) by the model

| 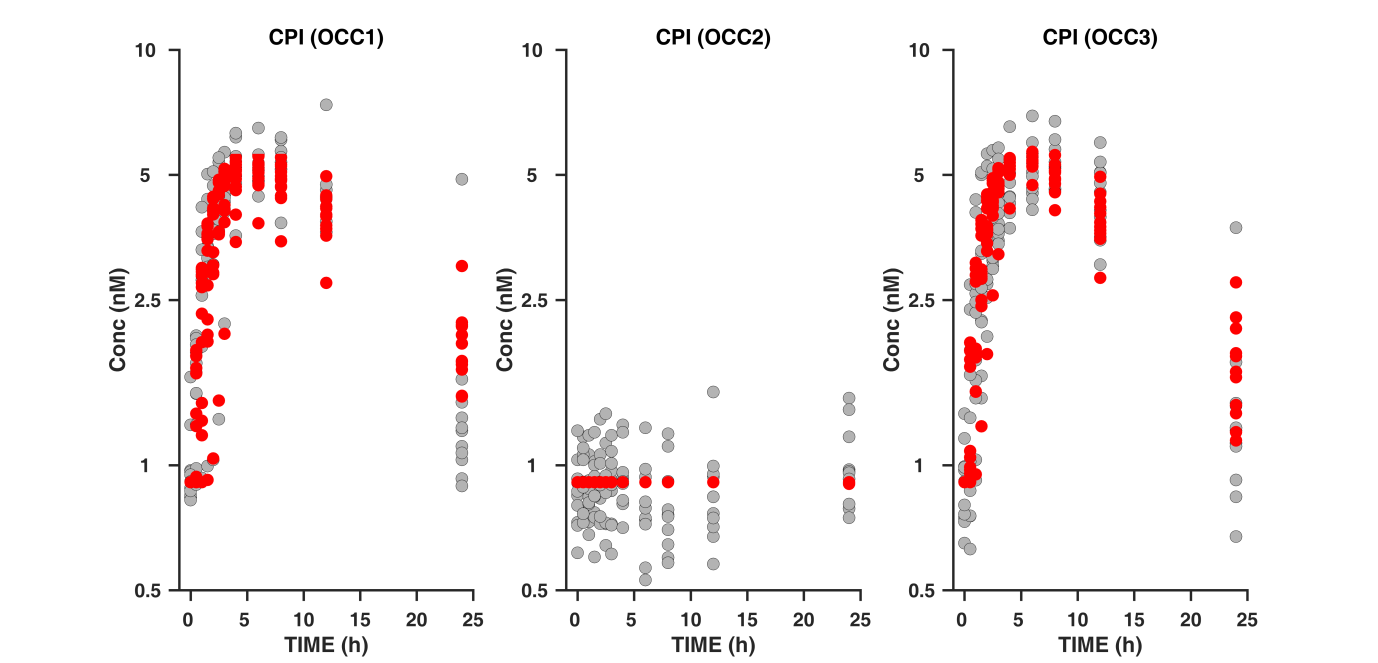 |
| --- |
| 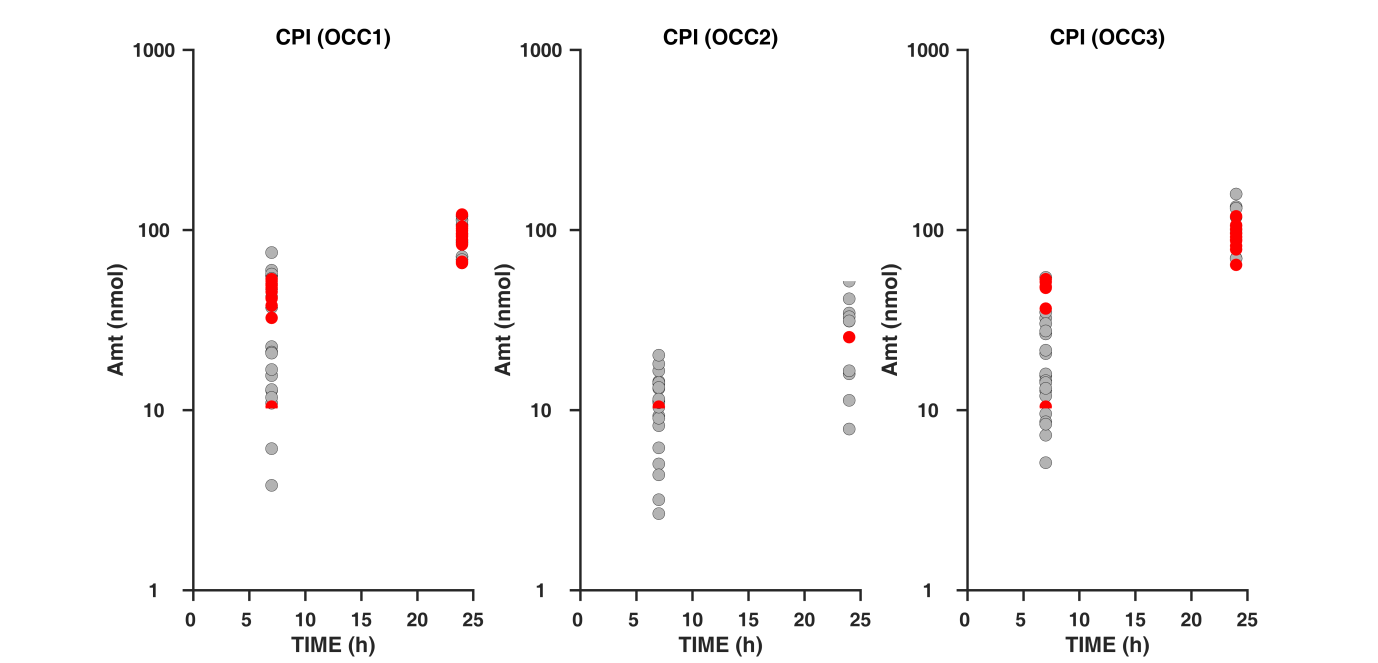 |

**Figure S9:** Observed plasma concentration data (Upper panel) and urine data (Lower panel) (grey circles) and the fitted population prediction (red circles) of CPI by the model stratified by occasion (OCC1, 2 and 3)


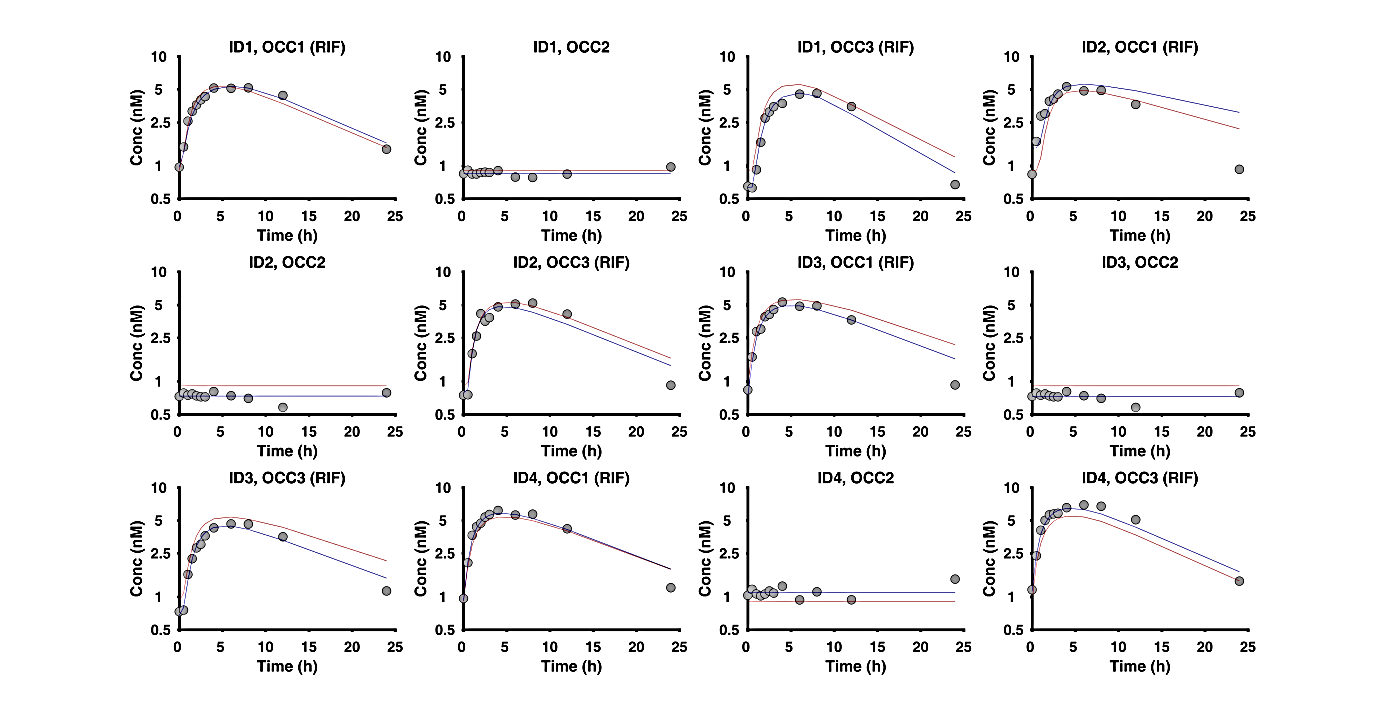

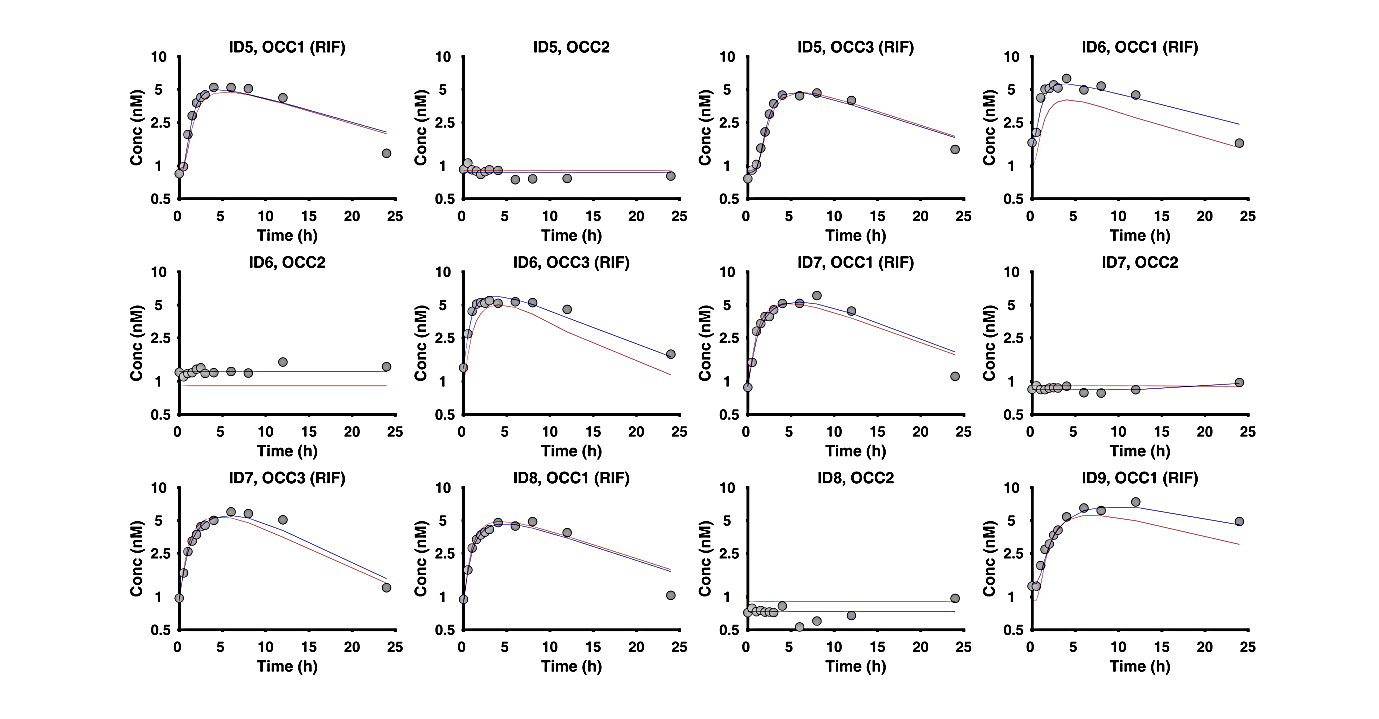


**
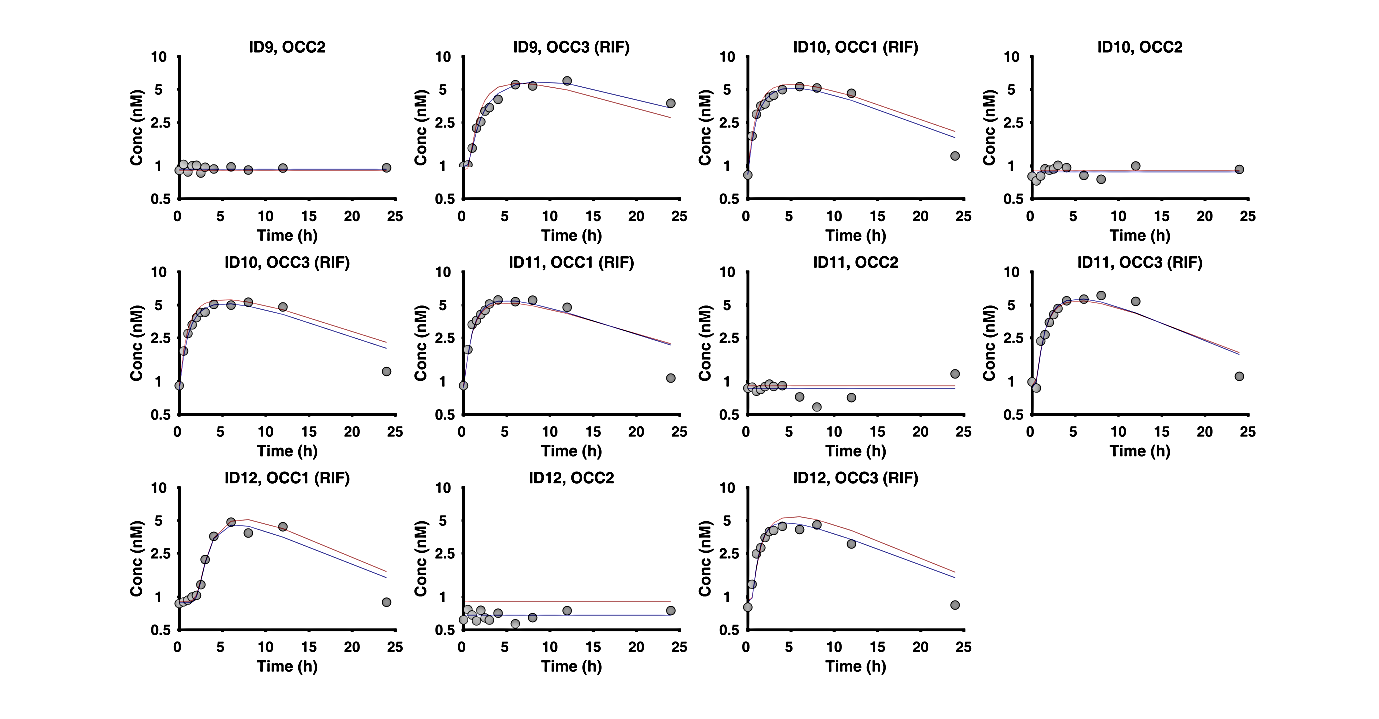
Figure S10:** Individual plasma concentration data of CPI (grey circles) and the fitted population (red line) and individual prediction (blue line) by the model.


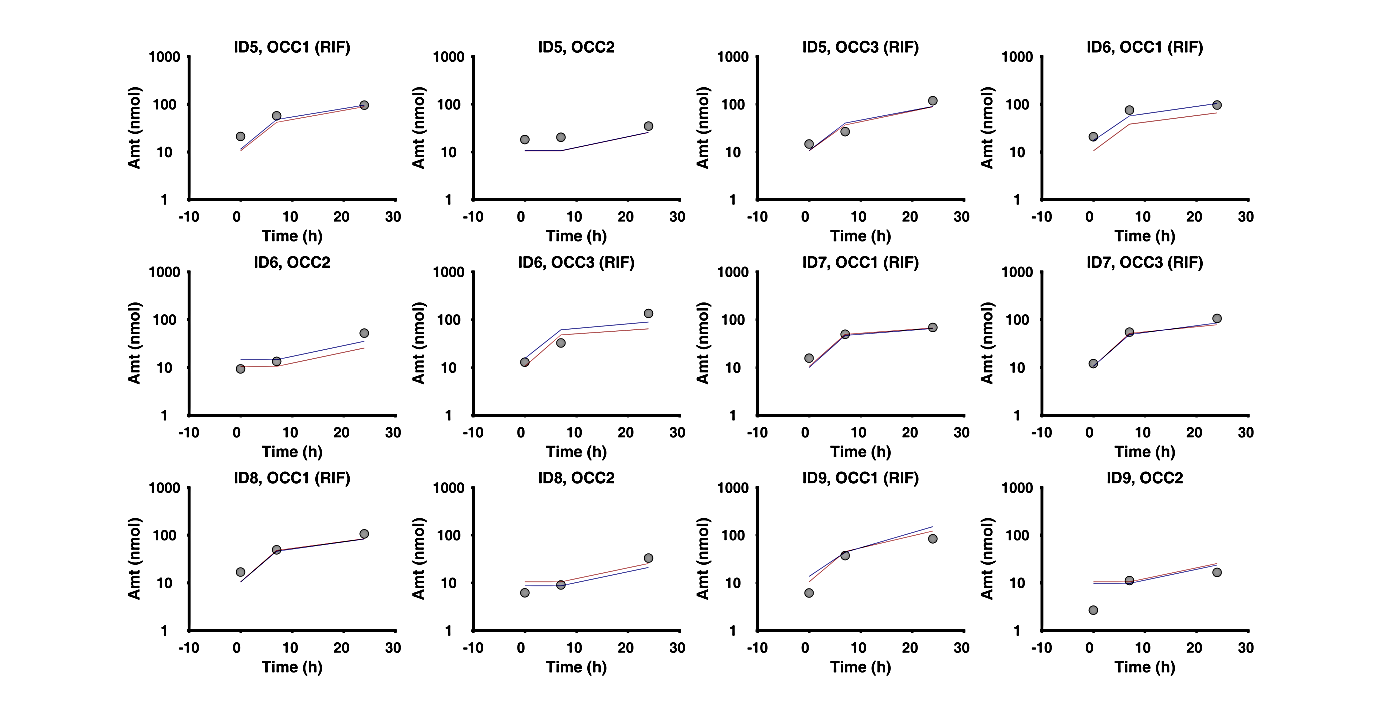

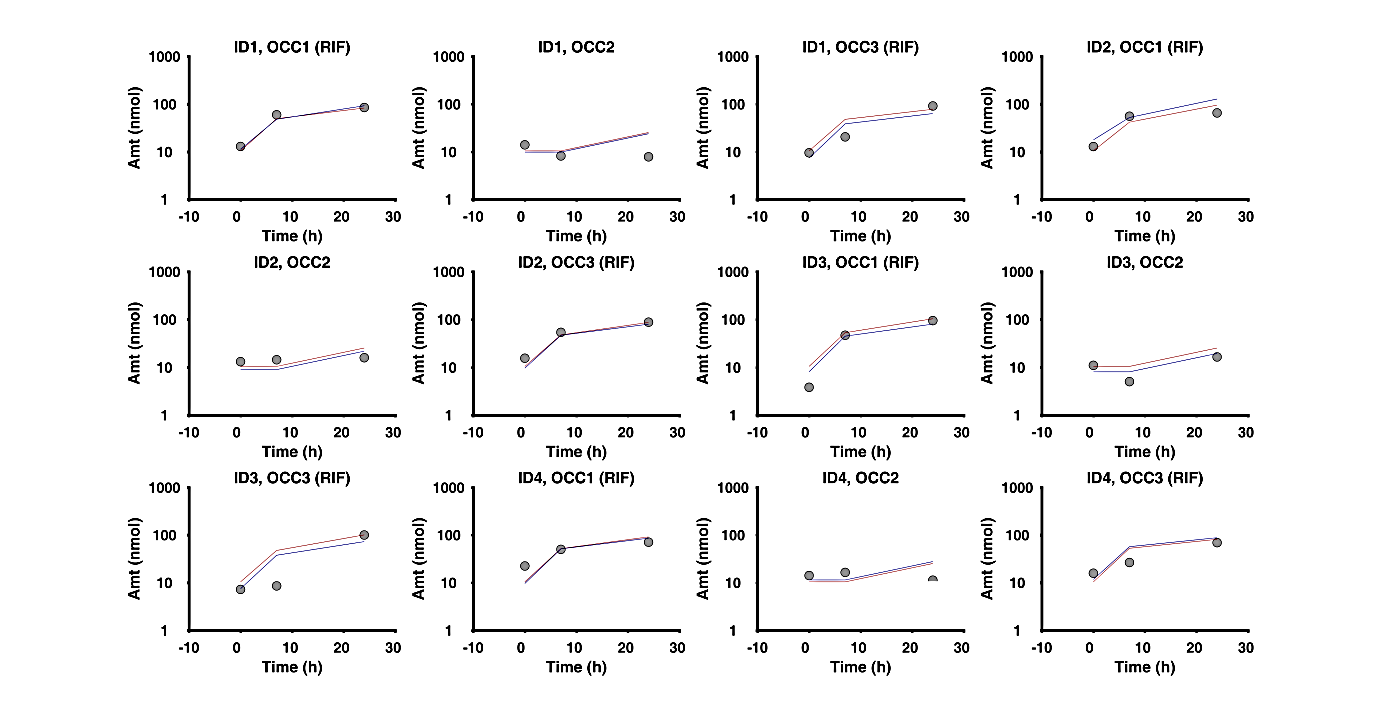


**
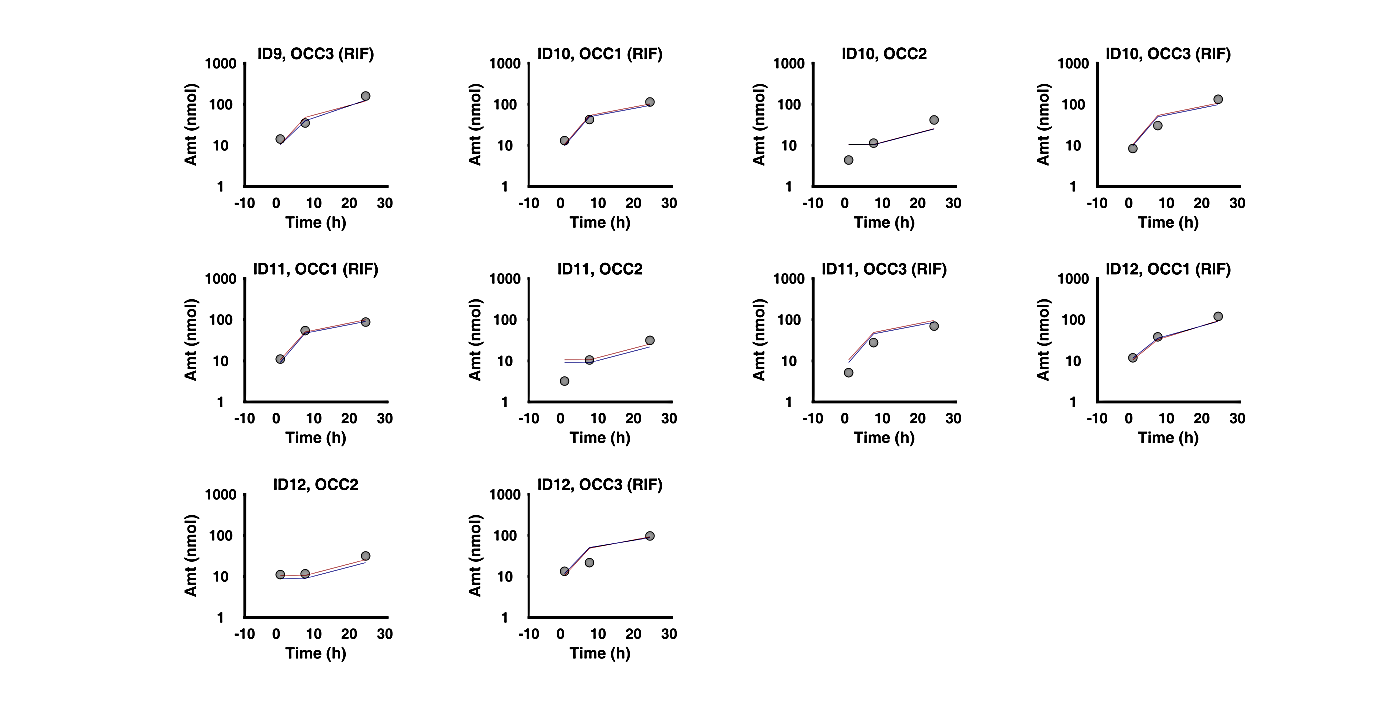
**

**Figure S11:** Individual urine amount data of CPI (grey circles) and the fitted population (red line) and individual prediction (blue line) by the model.


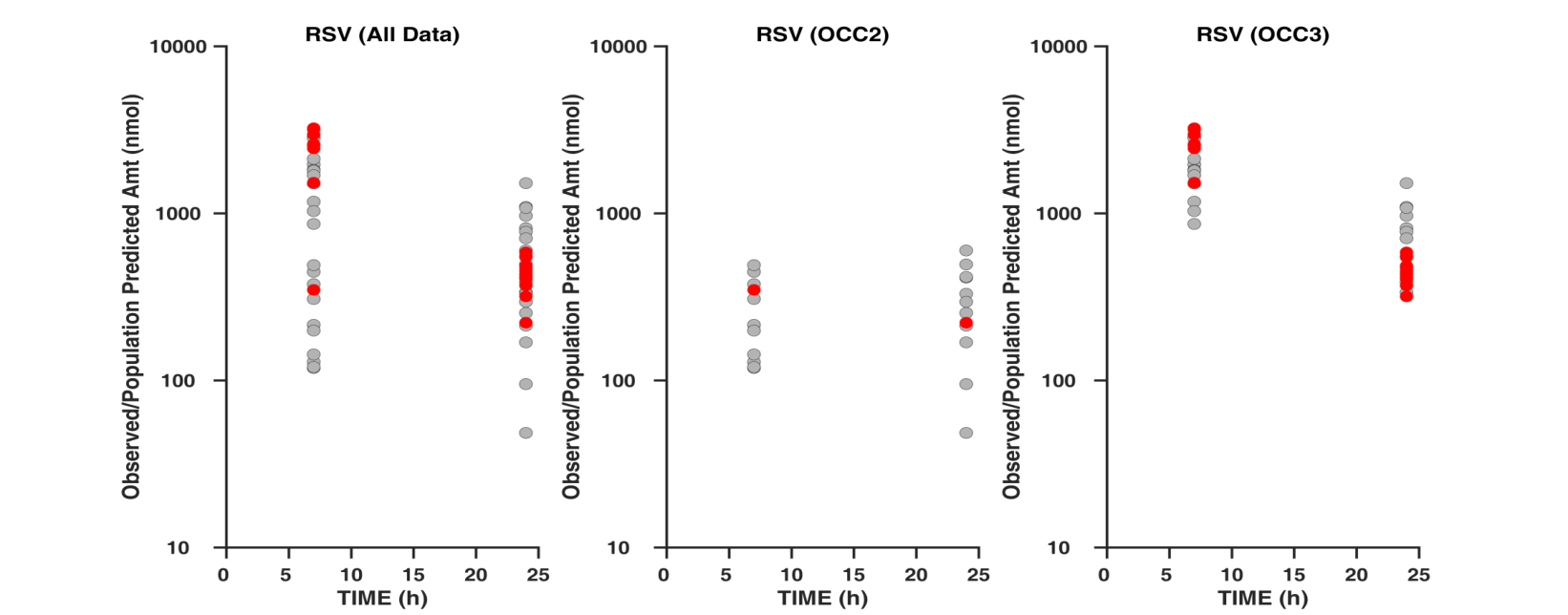

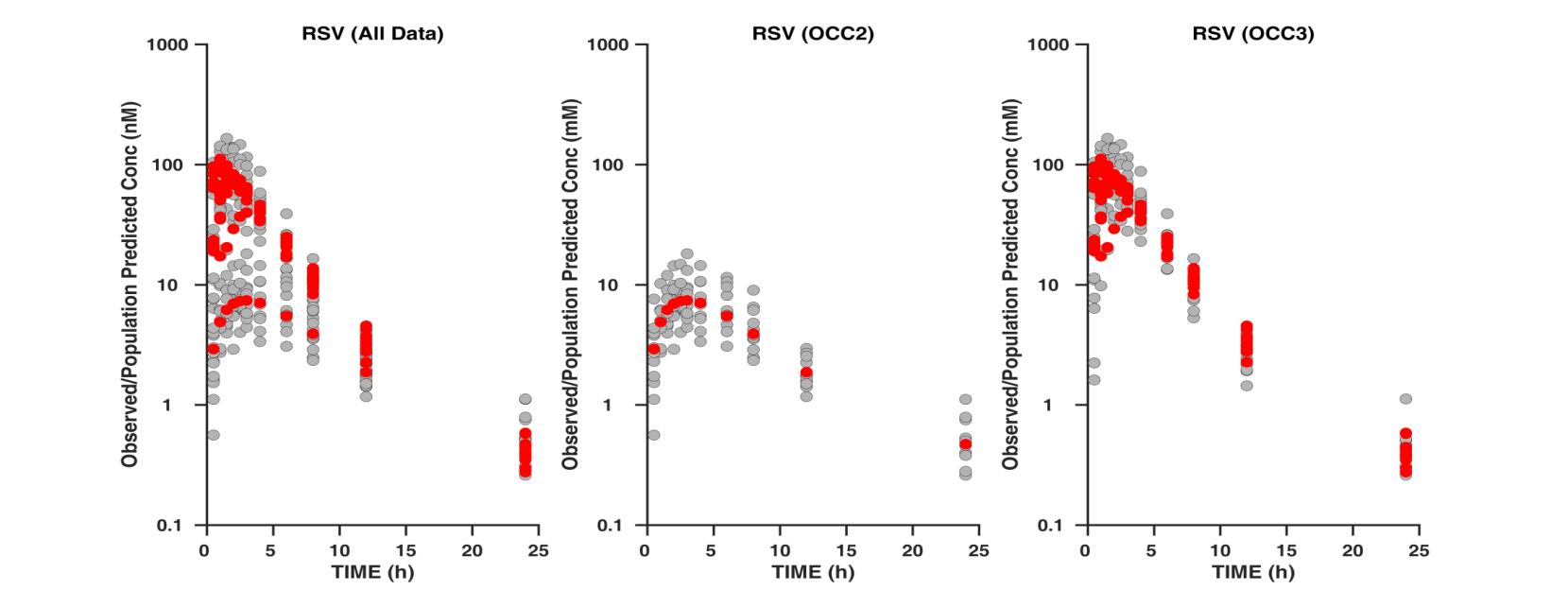


**Figure S12:** Observed plasma concentration data (Upper panel) and urine data (Lower panel) (grey circles) and the fitted population prediction (red circles) of RSV by the model stratified by occasion (OCC 2 and 3)


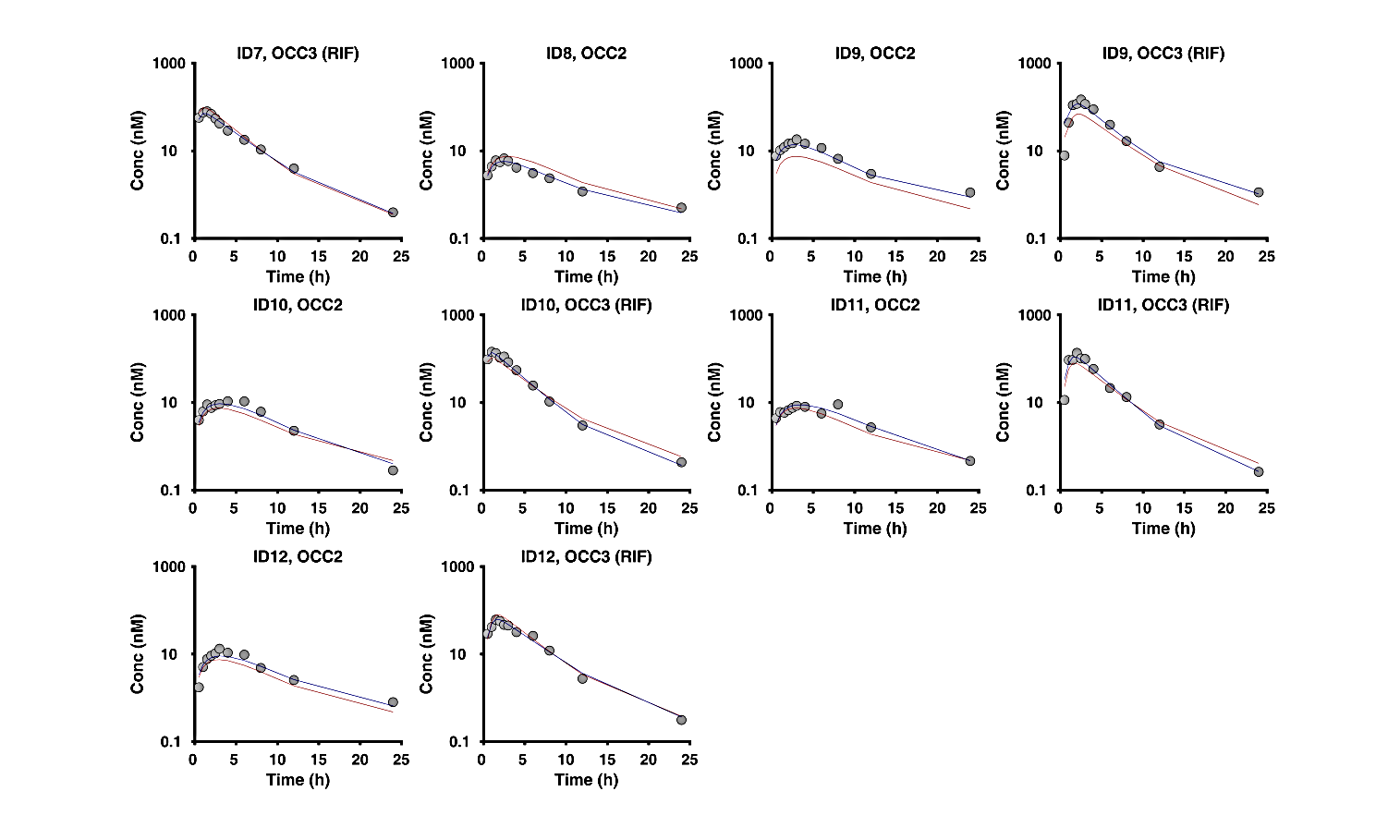

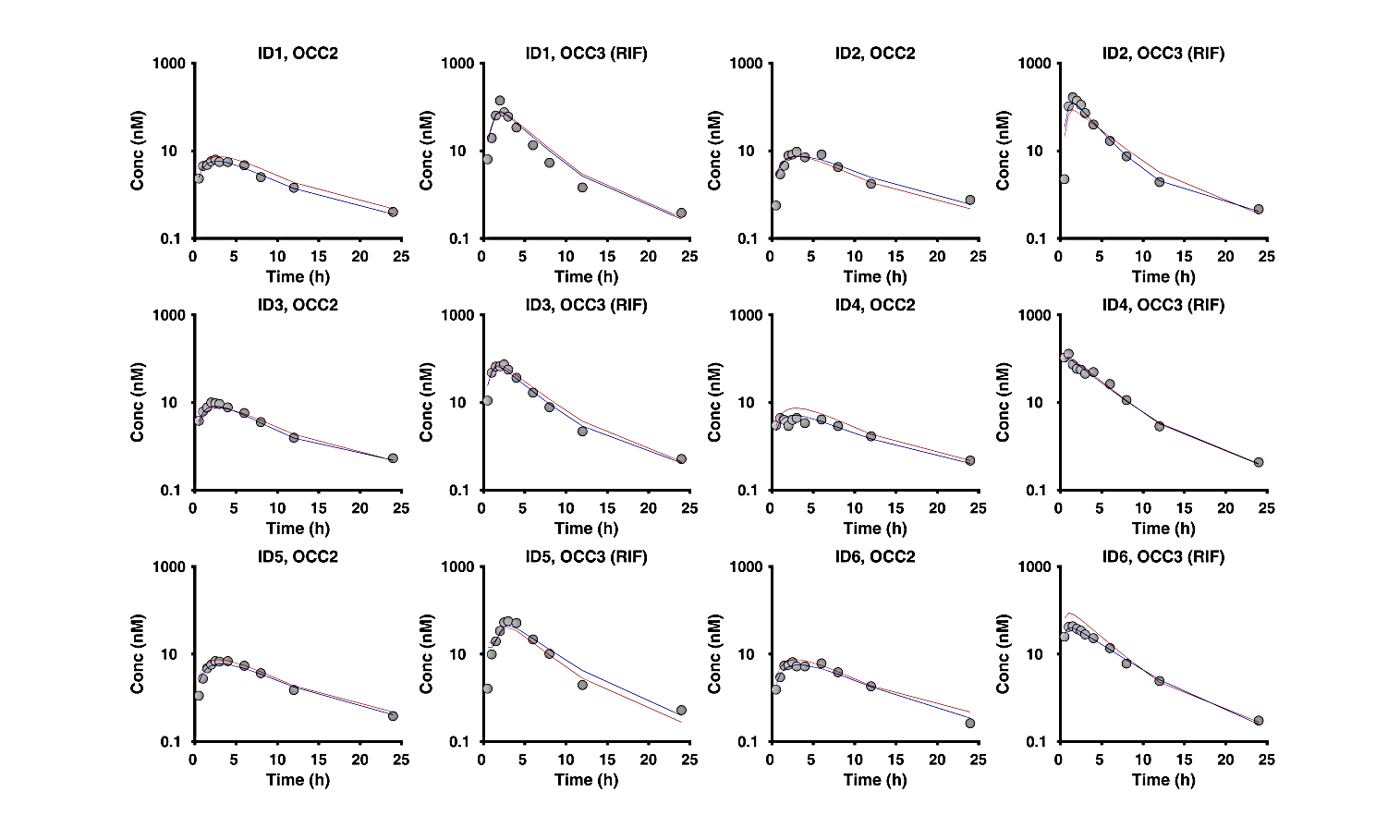


**Figure S13**: Individual plasma concentration data of RSV (grey circles) and the fitted population (red line) and individual prediction (blue line) by the model.


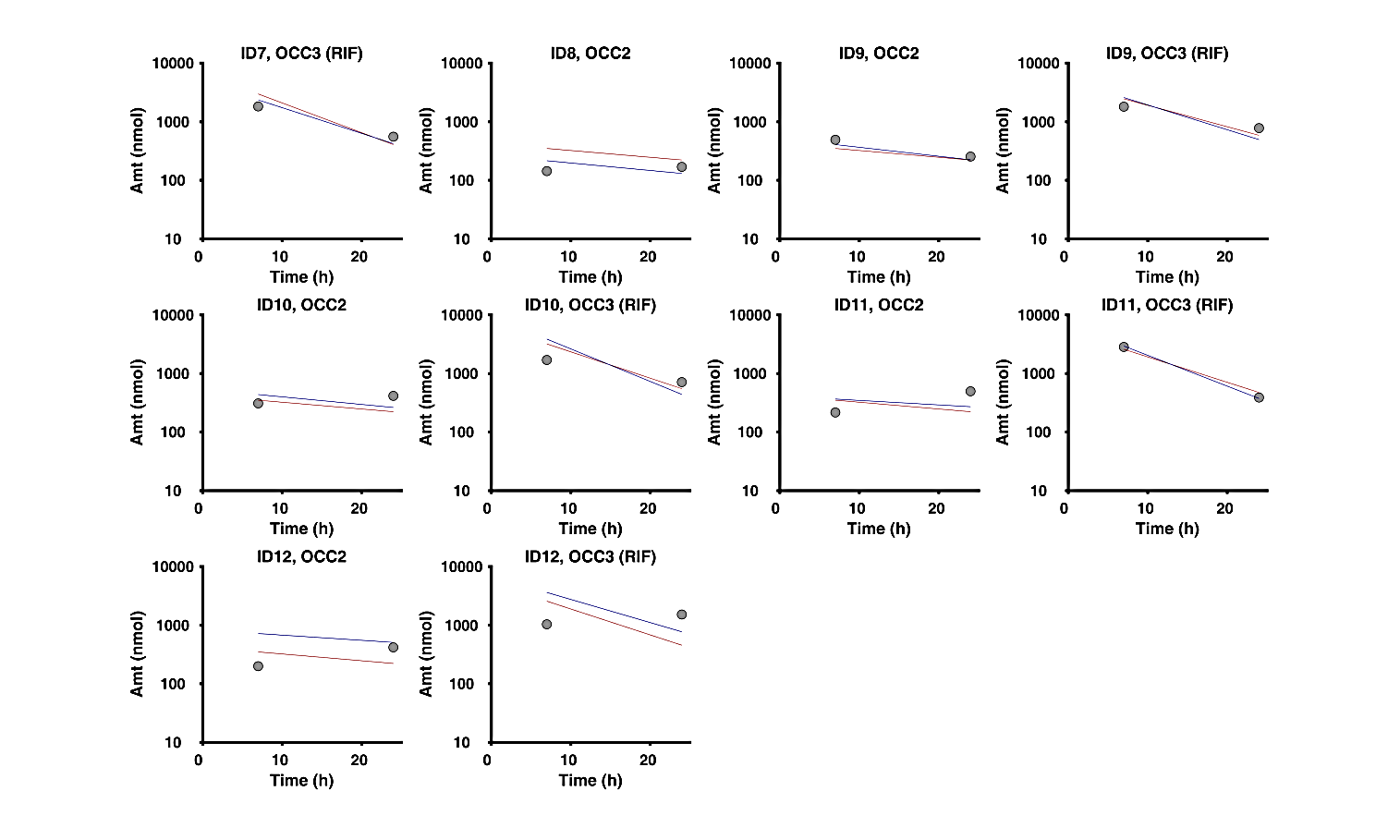

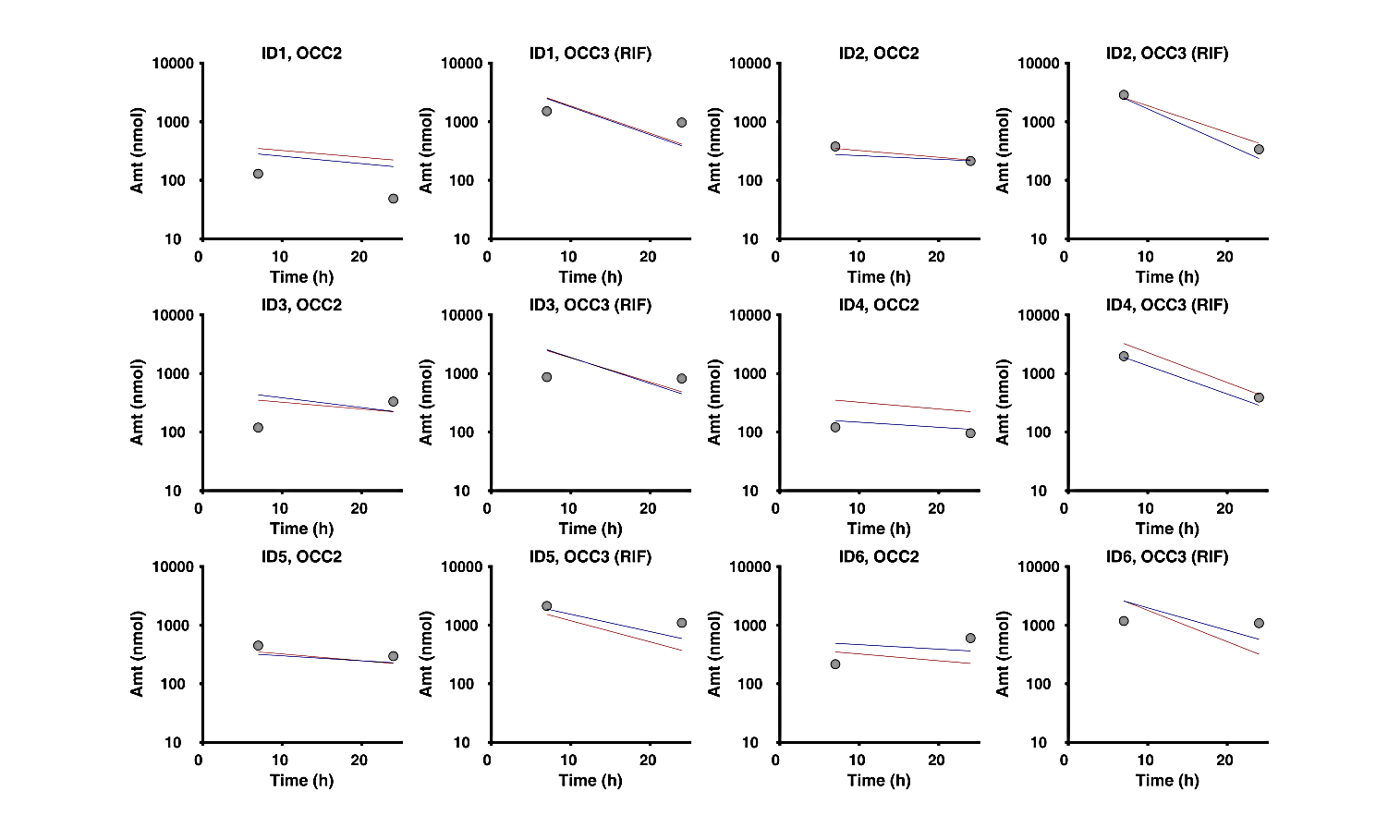


**Figure S14:** Individual urine amount data of CPI (grey circles) and the fitted population (red line) and individual prediction (blue line) by the model


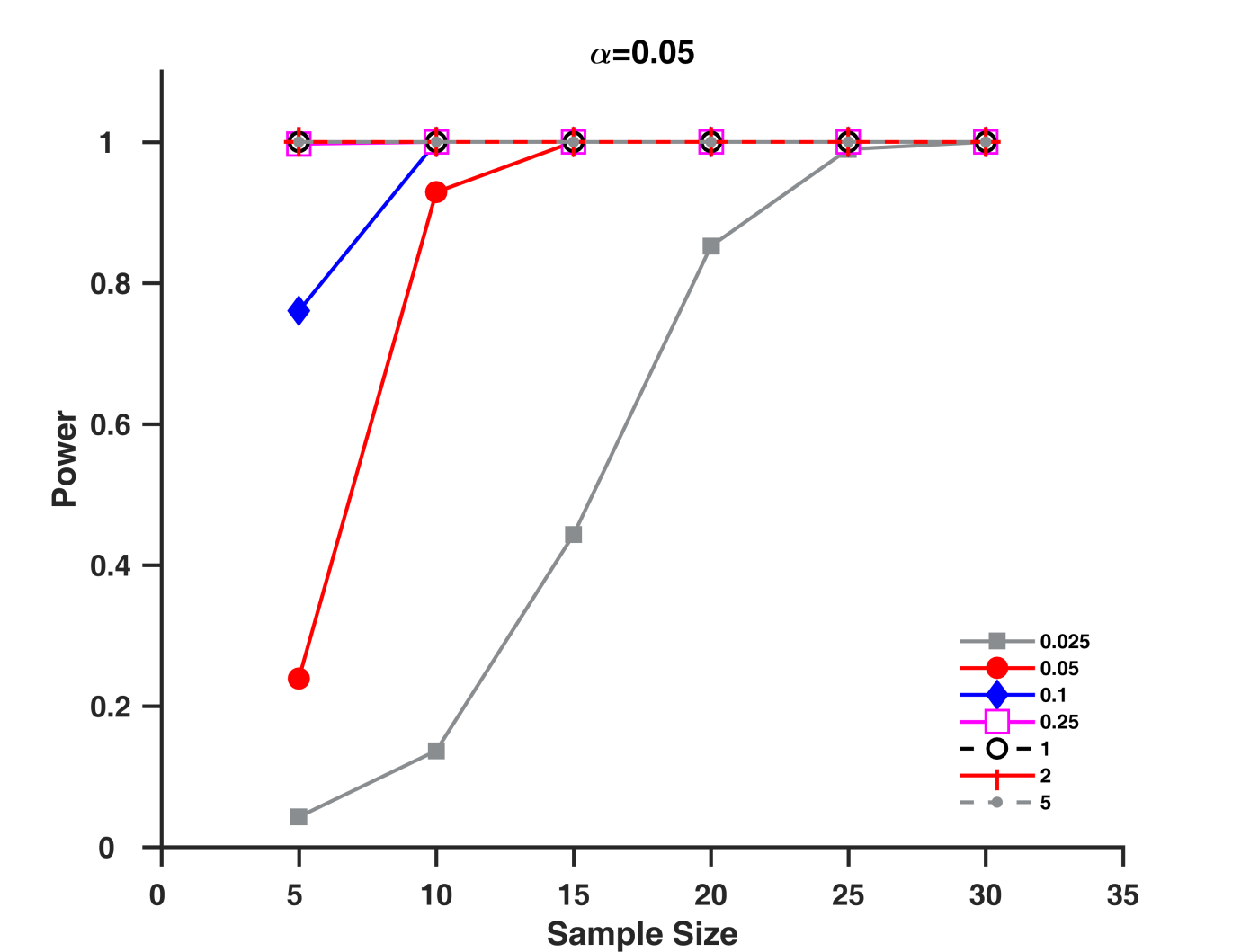


**Figure** **S15**: Power curves at two significance level (α=0.05) for the different hypothetical I/Ki ratios based on one-sample paired t-test of the ratio of logarithmic transformed AUC.

**Table S6:** Summary of rosuvastin interactions with known OATP inhibiors and the resulting fold change in AUC

| Severity of DDI | Inhibitor | Dose of Inhibitor (mg) | Dose of Rosuvastatin (mg) | Fold Change in AUC | Ref |
| --- | --- | --- | --- | --- | --- |
| Weak (AUCR<2) | Asunaprevir | 200 | 10 | 1.41 | ([14](#_ENREF_14)) |
|  | Clarithromycin | 500 | 0.025 | 1.56 | ([15](#_ENREF_15)) |
|  | Grazoprevir | 200 | 10 | 1.59 | ([16](#_ENREF_16)) |
|  | Itraconazole | 200 | 0.025 | 1.78 | ([15](#_ENREF_15)) |
| Moderate (2<AUCR<5) | Paritaprevir | 150 | 10 | 2.6 | ([17](#_ENREF_17)) |
|  | Velpatasvir | 100 | 10 | 2.69 | ([18](#_ENREF_18)) |
|  | Simeprevir | 150 | 10 | 2.8 | ([19](#_ENREF_19)) |
|  | Rifampicin | 600 | 5 | 4.7 | ([20](#_ENREF_20)) |
| Strong  (AUCR>5) | Cyclosporine | 75-200 | 10 | 7.1 | ([21](#_ENREF_21)) |

**References**

(1) Menochet, K., Kenworthy, K.E., Houston, J.B. & Galetin, A. Use of Mechanistic Modelling to Assess Inter-Individual Variability and Inter-species Differences in Active Uptake in Human and Rat Hepatocytes. *Drug Metab Dispos* **40**, 1744-56 (2012).

(2) Poirier, A. *et al.* Design, data analysis, and simulation of in vitro drug transport kinetic experiments using a mechanistic in vitro model. *Drug Metab Dispos* **36**, 2434-44 (2008).

(3) Bednarczyk, D. & Boiselle, C. Organic anion transporting polypeptide (OATP)-mediated transport of coproporphyrins I and III. *Xenobiotica* **46**, 457-66 (2016).

(4) Shen, H. *et al.* Comparative Evaluation of Plasma Bile Acids, Dehydroepiandrosterone Sulfate, Hexadecanedioate and Tetradecanedioate with Coproporphyrins I and III as Markers of OATP Inhibition in Healthy Subjects. *Drug Metab Dispos*, (2017).

(5) Takehara, I. *et al.* Investigation of Glycochenodeoxycholate Sulfate and Chenodeoxycholate Glucuronide as Surrogate Endogenous Probes for Drug Interaction Studies of OATP1B1 and OATP1B3 in Healthy Japanese Volunteers. *Pharm Res*, (2017).

(6) Pahwa, S. *et al.* Pretreatment With Rifampicin and Tyrosine Kinase Inhibitor Dasatinib Potentiates the Inhibitory Effects Toward OATP1B1- and OATP1B3-Mediated Transport. *J Pharm Sci* **106**, 2123-35 (2017).

(7) Izumi, S. *et al.* Substrate-dependent inhibition of organic anion transporting polypeptide 1B1: comparative analysis with prototypical probe substrates estradiol-17beta-glucuronide, estrone-3-sulfate, and sulfobromophthalein. *Drug Metab Dispos* **41**, 1859-66 (2013).

(8) Prueksaritanont, T. *et al.* Pitavastatin is a more sensitive and selective organic anion-transporting polypeptide 1B clinical probe than rosuvastatin. *Br J Clin Pharmacol* **78**, 587-98 (2014).

(9) Shen, H. *et al.* Cynomolgus monkey as a potential model to assess drug interactions involving hepatic organic anion transporting polypeptides: in vitro, in vivo, and in vitro-to-in vivo extrapolation. *J Pharmacol Exp Ther* **344**, 673-85 (2013).

(10) Izumi, S. *et al.* Investigation of the impact of substrate selection on in vitro organic anion transporting polypeptide 1B1 inhibition profiles for the prediction of drug-drug interactions. *Drug Metab Dispos* **43**, 235-47 (2015).

(11) Vildhede, A. *et al.* Hepatic uptake of atorvastatin: influence of variability in transporter expression on uptake clearance and drug-drug interactions. *Drug Metab Dispos* **42**, 1210-8 (2014).

(12) Wilkins, J.J. *et al.* Population pharmacokinetics of rifampin in pulmonary tuberculosis patients, including a semimechanistic model to describe variable absorption. *Antimicrob Agents Chemother* **52**, 2138-48 (2008).

(13) McColl, K.E., Thompson, G.G., el Omar, E., Moore, M.R., Park, B.K. & Brodie, M.J. Effect of rifampicin on haem and bilirubin metabolism in man. *Br J Clin Pharmacol* **23**, 553-9 (1987).

(14) Eley, T. *et al.* Organic anion transporting polypeptide-mediated transport of, and inhibition by, asunaprevir, an inhibitor of hepatitis C virus NS3 protease. *Clin Pharmacol Ther* **97**, 159-66 (2015).

(15) Prueksaritanont, T. *et al.* Validation of a microdose probe drug cocktail for clinical drug interaction assessments for drug transporters and CYP3A. *Clin Pharmacol Ther* **101**, 519-30 (2017).

(16) FDA. Zepatier Highlights of Prescribing Information. *https://wwwaccessdatafdagov/drugsatfda_docs/label/2016/208341s000lblpdf*, (2016).

(17) Menon, R.M. *et al.* Drug-drug interaction profile of the all-oral anti-hepatitis C virus regimen of paritaprevir/ritonavir, ombitasvir, and dasabuvir. *J Hepatol* **63**, 20-9 (2015).

(18) FDA. Epclusa Highlights of Prescribing Information. *https://wwwaccessdatafdagov/drugsatfda_docs/label/2016/208341s000lblpdf*, (2016).

(19) FDA. Olysio Highlights of Prescribing Information. *https://wwwaccessdatafdagov/drugsatfda_docs/label/2013/205123s001lblpdf*, (2013).

(20) Lai, Y. *et al.* Coproporphyrins in Plasma and Urine Can Be Appropriate Clinical Biomarkers to Recapitulate Drug-Drug Interactions Mediated by Organic Anion Transporting Polypeptide Inhibition. *J Pharmacol Exp Ther* **358**, 397-404 (2016).

(21) Simonson, S.G. *et al.* Rosuvastatin pharmacokinetics in heart transplant recipients administered an antirejection regimen including cyclosporine. *Clin Pharmacol Ther* **76**, 167-77 (2004).
